# Genomic and functional analyses unveil the response to hyphal wall stress in *Candida albicans* cells lacking β(1,3)-glucan remodeling

**DOI:** 10.1186/s12864-016-2853-5

**Published:** 2016-07-02

**Authors:** Genny Degani, Enrico Ragni, Pedro Botias, Davide Ravasio, Julia Calderon, Elena Pianezzola, Jose Manuel Rodriguez-Peña, Maria Antonietta Vanoni, Javier Arroyo, William A. Fonzi, Laura Popolo

**Affiliations:** Dipartimento di Bioscienze, Università degli Studi di Milano, Via Celoria 26, 20133 Milano, Italy; Unidad de Genómica, CAI de Genómica y Proteómica, UCM, Madrid, Spain; Departamento de Microbiologia II, Facultad de Farmacia, Universidad Complutense de Madrid, Madrid, Spain; Department of Microbiology and Immunology, Georgetown University, Washington, D.C USA; Present address: Unit of Cell therapy and Cryobiology, Fondazione IRCCS Cà Granda, Ospedale Maggiore Policlinico, Milano, Italy; Present address: Evolva, Basel, Switzerland; Present address: Instituto de Biología Funcional y Genómica, Salamanca, Spain

**Keywords:** Hyphal growth, Cell wall, β(1,3)-glucan, Chitin, Cell integrity, Family GH72, MAP kinases

## Abstract

**Background:**

The cell wall is essential for the yeast to hypha (Y-H) transition that enables *Candida albicans* to invade human tissues and evade the immune system. The main constituent, β(1,3)-glucan, is remodeled by glucanosyltransferases of the GH72 family. Phr1p is responsible of glucan remodeling at neutral-alkaline pH and is essential for morphogenesis and virulence. Due to the pH-regulated expression of *PHR1*, the *phr1*Δ phenotype is manifested at pH > 6 and its severity increases with the rise in pH. We exploited the pH-conditional nature of a *PHR1* null mutant to analyze the impact of glucan remodeling on the hyphal transcriptional program and the role of chitin synthases in the hyphal wall stress (HWS) response.

**Results:**

In hyphal growth inducing conditions, *phr1*Δ germ tubes are defective in elongation, accumulate chitin, and constitutively activate the signaling pathways mediated by the MAP kinases Mkc1p, Cek1p and Hog1p. The transcriptional profiles revealed an increase of transcript levels for genes involved in cell wall formation (*CHS2* and *CHS8*, *CRH11*, *PGA23*, orf19.750, *RBR1*, *RBT4*, *ECM331*, *PGA6*, *PGA13*), protein *N*-glycosylation and sorting in the ER (*CWH8* and *CHS7)*, signaling (*CPP1, SSK2*), ion transport (*FLC2*, *YVC1*), stress response and metabolism and a reduced expression of adhesins. A transient up-regulation of DNA replication genes associated with entry into S-phase occurred whereas cell-cycle regulating genes (*PCL1*, *PCL2*, *CCN1, GIN4*, *DUN1*, *CDC28*) were persistently up-regulated. To test the physiological relevance of altered *CHS* gene expression, *phr1*Δ *chsx*Δ (x = 2,3,8) mutant phenotypes were analyzed during the Y-H transition. *PHR1* deletion was synthetic lethal with *CHS3* loss on solid M199 medium-pH 7.5 and with *CHS8* deletion on solid M199-pH 8. On Spider medium, *PHR1* was synthetic lethal with *CHS3* or *CHS8* at pH 8.

**Conclusions:**

The absence of Phr1p triggers an adaptive response aimed to reinforce the hyphal cell wall and restore homeostasis. Chs3p is essential in preserving *phr1*Δ cell integrity during the Y-H transition. Our findings also unveiled an unanticipated essential role of Chs8p during filamentation on solid media. These results highlight the flexibility of fungal cells in maintaining cell wall integrity and contribute to assessments of glucan remodeling as a target for therapy.

**Electronic supplementary material:**

The online version of this article (doi:10.1186/s12864-016-2853-5) contains supplementary material, which is available to authorized users.

## Background

*Candida albicans* is a medically important fungal pathogen that exhibits various morphological forms: yeast, hypha, pseudohypha and chlamydospore. As a commensal, *C. albicans* colonizes human *mucosae* and is a component of the oral fungal microbiome [1]. Its extraordinary ability to inhabit diverse niches of the human body is reflected in its adaptability to a wide range of ambient pH values and to changes in oxygen pressures, ion concentrations, and carbon sources [2, 3]. As an external envelope endowed with mechanical strength, the cell wall plays a primary role in determining cell shape and in maintaining cell integrity during morphological changes or osmotic shock. Additionally, the surface of the cell wall is positioned at the interface between the pathogen and host cells and thus mediates dynamic interactions crucial for pathogenesis. Whereas the yeast form is suitable for dissemination through the blood stream, the thin filamentous shape of hyphae is specialized for adhesion to epithelial and endothelial barriers, and penetration and invasion of the tissues below [4]. Genomic-scale expression studies have identified a number of signature genes induced by the yeast to hypha (Y-H) transition [5–7].

Hypha formation requires a coupling between the polarity machinery and the biogenesis of the wall in order to drive growth at the tip of the germ tube. Cell wall formation requires synthesis and assembly of two glucose polymers, β(1,3)-glucan, the most abundant, and β(1,6)-glucan, and synthesis and incorporation of mannoproteins. Most mannoproteins are modified by attachment of glycosylphosphatidylinositol (GPI) and are localized in the plasma membrane but can be further processed and covalently linked to cell wall glucan (reviewed in [8]). Chitin is a minor constituent but it is crucial for the formation of the septum and for the structural integrity of the wall. In the extracellular compartment, a branched β(1,3)glucan-chitin core structure is created and decorated by links between chitin and β(1,6)-glucan or trimmed GPI-mannoproteins, the latter forming the “brush-like” surface layer which functions as a permeability barrier and adhesive surface [9].

Among the extracellular enzymes orchestrating cell wall assembly, β(1,3)-glucanosyltransferases of family GH72 play a primary role. These enzymes internally cleave a donor glucan chain and attach a portion of the donor to an acceptor glucan in β(1,3)-linkage, thus lengthening one chain at the expense of the other [10]. Multigene families encoding redundant enzymes are present in all fungal species so far analyzed and are essential for viability in many species [11–14]. *C. albicans* has a family of five GH72-encoding genes: *PHR1, PHR2, PHR3*, *PGA4* and *PGA5*. Phr1p and Phr2p are highly similar to *Saccharomyces cerevisiae* Gas1p, they share the same activity in vitro and *PHR1* complements *gas1*Δ mutation [15, 16]. Pga4p is most similar to the paralog of *Sc*Gas1p, the auxiliary *Sc*Gas5p, and was previously detected in proteomic analysis of *C. albicans* cells [17]*.* Since *PGA4* is transiently up-regulated in infection models and its deletion does not convey any obvious phenotype, it has been suggested that it may have some subtle roles in specific conditions [18]. Recent evidence from our laboratories indicate that Pga4p is an inactive enzyme and ectopic expression of *PGA4* is unable to complement *gas1*Δ mutation in *S. cerevisiae* (W. Fonzi, unpublished results and [16]). Phr3p and Pga5p are homologous to the sporulation-specific *Sc*Gas4p and *Sc*Gas2p, respectively [16]. The significance and the role of *PHR3* and *PGA5* are still unknown but the transcript level of both is very low or undetectable [18]. Thus, Phr1p and Phr2p appear to be the only active β(1,3)-glucanosyltransferases in *C. albicans*.

The expression of *PHR1* and *PHR2* is regulated in response to ambient pH. *PHR1* is expressed when the external pH is higher than 6 both in yeast and hyphal cells. It is repressed in acidic conditions where it is replaced by *PHR2* which exhibits the opposite expression pattern [19, 20]. Accordingly, the pH optima of recombinant Phr1p and Phr2p are consistent with their pattern of expression [16]. *PHR1* is also transcriptionally induced in response to heat stress, to treatments with the antifungal drug caspofungin and during infection [21–24]. Consistent with its enzymatic activity, Phr1p localizes to sites of cell wall formation such as the site of bud emergence, the periphery of the bud, the septum, the tip of the germ tube, and the hyphal apex and septa [25]. At the septum, Phr1p may convert polydisperse glucan to high molecular weight as shown for *Sc*Gas1p at the mother-bud neck [26, 27]. At pH 7.5 and 25 °C, loss of Phr1p affects cell wall composition, with a reduction of β(1,3)-glucan content, an increase of chitin and a loss of cell wall compactness [25, 28]. The phenotype is more severe if the pH is raised from 7.5 to 8, where greatly enlarged vacuoles and a rounder cell shape are present [20]. Moreover, *PHR1* null mutants are avirulent in an animal model of systemic infection and in a model of experimental keratomycosis [29, 30]. A *PHR1* null mutant is unable to invade in vitro reconstituted epithelia and has a reduction in adhesion, two fundamental processes for the establishment of fungal infections [31].

By exploiting the pH-conditional nature of the *PHR1* null mutant, which manifests its defects in wall assembly only upon a shift to neutral-alkaline pH, we monitored the severity of the hyphal wall stress (HWS) response and analyzed the impact of defective glucan remodeling on the transcription profile during the induction of hyphal growth. Among the classes of transcriptionally induced genes, we experimentally explored the physiological relevance of *CHS2* and *CHS8* and tested the effect of DNA replication genes on entry in S-phase. The results underline the striking capability of *C. albicans* to adjust its physiological systems to generate an optimized adaptive response and the unanticipated relevant role of *CHS8* in protecting cells from lysis beside the compensatory function of *CHS3* that is common to many fungal species.

## Results

### Time-course microarray analysis upon induction of hyphal growth in wild type and *PHR1* null mutant

**Fig. 1:**
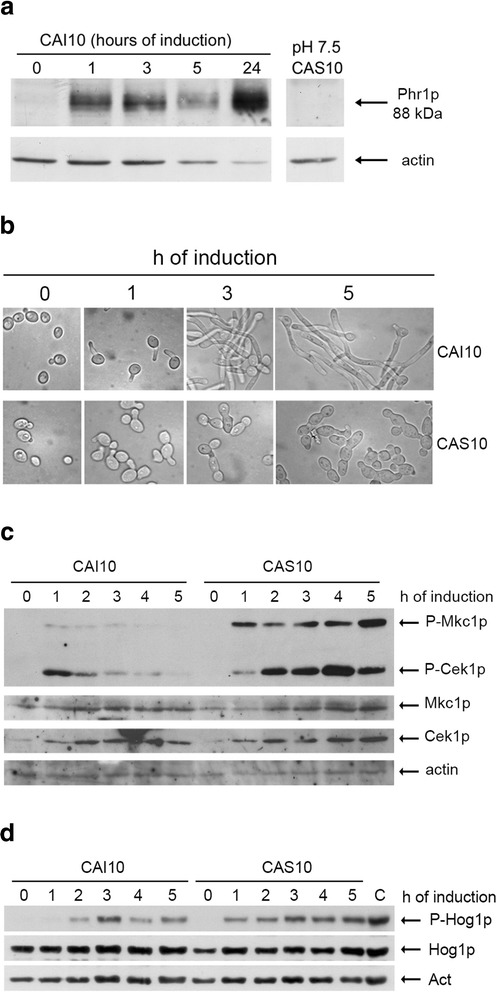
Lack of Phr1p affects morphology and constitutively activates MAP kinase signaling pathways during induction of hyphal growth in *C. albicans*. **a** Western blot analysis of Phr1p accumulation. At time zero, blastospores of the wild type (CAI10) were shifted to M199-150 mM HEPES buffered at pH 7.5 at 37 °C. Total protein extracts (125 μg) were analyzed using cross-reacting anti-*Sc*Gas1p antibodies and anti-actin mAb. The extract from *phr1*Δ (CAS10) corresponds to 5 h after the shift and shows the absence of Phr1p. **b** Morphology of the indicated strains during induction of hyphal growth. **c** Activation of the Map kinases Mkc1p and Cek1p during induction of hyphal growth. Western blot analysis was performed as described in Methods. Mkc1p and Cek1p migrated as bands of ~ 59 kDa and 48 kDa respectively. Their identity was confirmed using *MKC1* and *CEK1* null mutants (data not shown). **d** Activation of Hog1p during induction of hyphal growth. Sample “C” represents a control of an extract from vegetative CAI10 cells exposed for 2 min to hyperosmotic shock (0.6 M KCl in minimal medium)

To induce hyphal growth, a pH/temperature regimen was used. Blastospores of the wild type strain (CAI10) and *phr1*Δ mutant (CAS10) were transferred to buffered M199, pH 7.5 at 37 °C. In accord with the stronger morphological defect at pH 8, the percentage of dead *phr1*Δ cells at 5 h, measured by methylene blue (MB) staining, was 15 % compared to about 5 % at pH 7.5 and therefore the latter condition was used for microarray analysis. Phr1p was absent in blastospores at the moment of the shift to pH 7.5 and accumulated in CAI10 cells as a glycosylated 88 kDa-polypeptide that was absent in CAS10 (*PHR1*−/^−^) as expected (Fig. 1a) [16]. Cells started to form germ tubes at 45 min and the percentage of germ tubes increased with time reaching 50 % at 1 h and 80-90 % by 3 h after the shift. As shown in Fig. 1b, at time zero the morphology of CAI10 and CAS10 was indistinguishable. At subsequent time points, CAI10 produced elongated hyphae whereas CAS10 germ tubes remained short and enlarged, with wide septa and with swollen apical compartments. At time zero, the two strains had the same chitin content [8.80 ± 0.66 and 8.50 ± 0.88 μg *N*-acetylglucosamine (GlcNac)/mg dry weight of cells in CAI10 and CAS10, respectively] supporting the observation that *PHR1* deletion does not confer a detectable phenotype in non-inducing conditions. After 5 hours, chitin level increased to 11.70 ± 1.07 GlcNac/mg d.w. of cells in CAI10, in agreement with the known accumulation of chitin in hyphae although the increase was less than that seen during serum induction. Chitin accumulation was greater in CAS10 cells, 16.40 ± 1.50 GlcNac/mg d.w. of cells. This ~ 40 % extra chitin could be ascribed to the presence of hyphal wall stress (HWS) caused by the lack of Phr1p activity.

To assess the onset of HWS, we monitored the dual phosphorylation of Mkc1p and Cek1p, the two MAP kinases that become activated upon treatment with cell wall perturbing agents and have been implicated in morphogenesis (Mkc1p), in the Y-H transition (Cek1p), invasive growth, cell wall biogenesis and virulence [32–34]. As shown in Fig. 1c, phosphorylation of the two kinases in wild type cells was detected at 1 h and then steadily decreased indicating that HWS was transient and hyphal wall integrity was rapidly restored. In contrast, in the mutant activation of both kinases was stronger and persisted for several hours. We also monitored the evolutionarily-conserved Hog1p MAP kinase, which is involved in cell wall remodeling in response to hyperosmotic and oxidative stresses [35]. Interestingly, in the wild type the phosphorylated form of Hog1p was detectable from 2 hours to 5 h with a peak at 3 h (Fig. 1d). In contrast, in the *phr1* mutant Hog1p activation occurred earlier, by 1 h, and progressively increased at subsequent time points (Fig. 1d). The constitutive activation of Mkc1p, Cek1p and Hog1p kinases indicates that *phr1*Δ mutant is subjected to severe HWS. Interestingly, a series of other experiments indicated that Hog1p phosphorylation in the wild type has an oscillatory behavior, but this aspect was not further investigated in this work (unpublished data).

**Fig. 2:**
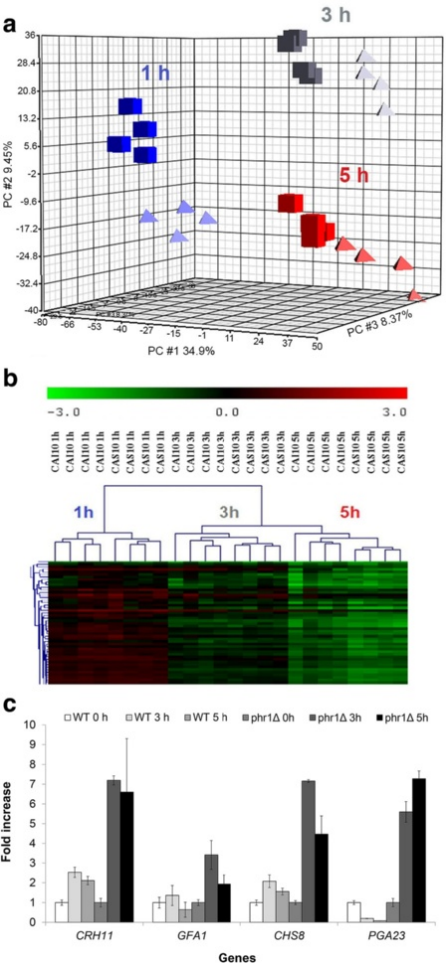
Global comparison of the transcriptional profile of wild type and *phr1*Δ cells during induction of hyphal growth. **a** Principal component analysis. Each of the four blocks for each strain represents one of the sets of hybridization results. The median of ratios was used. (▲), CAI 10; (■) CAS10. **b** Hierarchical cluster analysis of gene expression. The dendogram plot, illustrated a portion of the complete analysis, is shown. **c** Expression levels of the indicated genes determined by qRT-PCR. The data of each strain were normalized to time zero, set equal to 1. Data are mean values and bars indicate the standard deviations of three replicates of the same RNA (*n* = 3). Similar results were obtained in two independent biological replicates

To characterize the pattern of gene expression during hyphal development we performed DNA microarray analysis of strains CAI10 and CAS10 at 1, 3 and 5 h. Principal component analysis showed a clear separation between the wild type and mutant strains at each time point and underlined the high reproducibility of the biological replicas (Fig. 2a). Moreover, hierarchical clustering indicated that for both strains changes at 3 h and 5 h clustered tighter than at 1 h (Fig. 2b). Additional file 1 shows genes that were up- (≥2 fold) or down–regulated (≥2-fold) with a False Discovery Rate (FDR) ≤ 0.05 both in the wild type and mutant strains. This threshold was chosen to ensure that the analysis was stringent but would not miss significant genes. Among the 6,346 probes, 536 genes were up-regulated at 1 h, 731 at 3 h and 887 at 5 h in the wild type whereas 725, 701 and 909 genes were up-regulated in the *phr1*Δ mutant. Moreover, 768, 819 and 999 were down regulated at least 2-fold in the wild type and 893, 839 and 1,072 in the mutant. Comparison of the data from wild type and mutant strains identified a common group of responsive genes (60 %, 66 % and 64 % up-regulated and 65 %, 77 % and 76 % down regulated at 1 h, 3 h and 5 h, respectively), suggesting that *phr1*Δ mutant cells trigger the hyphal expression program in spite of their failure to form hyphae.

The modulation of many genes reflects the extensive differentiation associated with induction of the hyphal program and, at least in the early stages, the shift from quiescence to growth and the change in culture conditions (media composition, pH and temperature). Up-regulated genes belonged to the functional categories expected for hyphal development: hyphal growth, pathogenesis, endoplasmic reticulum (ER)-Golgi transport, cell wall organization, Golgi-ER retrograde transport, ER-associated-protein degradation (ERAD) and biofilm formation. The typical signature up-regulated genes of hyphal growth, such as *ECE1, SOD5, ALS3, RBT1, HWP1, HYR1 PRA1, SAP4, SAP5, SAP6* and the typical down-regulated genes *RHD1*, *TYE7, NRG1* were present [6]. *PHR1* itself is typically induced during hyphal growth and was up-regulated 1.4-fold at 1 h and 1.6-fold at 5 h in the wild type, in agreement with previous reports [6, 7]. As expected, *PHR1* mRNA was undetectable in CAS10. *PHR2* was repressed both in the wild type and in the mutant (Additional file 1).

### Global transcriptional response to HWS

To analyze the changes in gene expression caused by HWS, we used a relation factor (RF) representing the ratio between the expression ratio of each gene in the mutant and in the wild type (MUT/WT). Thus, RF reflects the effect of the *phr1*Δ mutation on the abundance of a transcript. We set a threshold of RF ≥ 2 to identify transcripts more abundant in the *phr1*Δ mutant than in the wild type (Class 1) and RF ≤ 0.5 for transcripts reduced in the *phr1*Δ mutant compared to the wild type (Class 2). Genes yielding a RF ≥ 2, but down-regulated both in the mutant and wild type, were not included in Class 1. Similarly, genes with RF ≤ 0.5 but up-regulated in both strains, were not included in Class 2. In total, Class 1 and Class 2 contained 115 and 87 genes with known functions, and 62 and 53 genes with unknown functions, respectively (Additional file 2 and Additional file 3).

Table 1 summarizes the functional categories of mutation-sensitive genes. Notably, in Class 1 the most abundant categories were “Cell wall” and “Metabolism”, indicating the cell’s need of reorganizing the cell surface and redirecting metabolism. The categories “DNA replication and repair”, “Chromatin and chromosomes” and “Cell cycle” were also affected, suggesting perturbations of the coordination between cell cycle events and morphogenesis. Class 2 contained many genes required for RNA processing and ribosome biogenesis primarily at 1 h.

**Table 1:** Functional categories and number of *phr1*Δ mutation-sensitive genes in Class1 and Class 2

	Functional category	1 h	3 h	5 h
Class 1				
	Unknown function	41	13	34
	Cell wall	13	14	11
	Metabolism	11	6	4
	DNA replication and repair	10	10	2
	Filamentous growth	9	3	8
	Oxido-reduction processes	6	2	2
	Chromatin and chromosomes	5	7	0
	Transcription	5	3	4
	Cell cycle	4	6	6
	Transport	4	2	3
	Cell polarity/cytoskeleton	3	1	2
	Signal transduction	2	2	3
	Protein folding/modification	2	1	4
	Stress response	1	2	2
	Adhesion	1	0	0
	RNA processing and biogenesis	1	0	0
	Pathogenesis	0	0	1
		118	72	86
Class 2				
	RNA processing and biogenesis	19	2	5
	Unknown function	15	17	35
	Transport	4	5	8
	Metabolism	3	6	14
	Transcription	3	3	9
	Protein folding/modification	2	4	3
	Oxido-reduction processes	1	1	6
	Cell wall	1	1	2
	Stress response	0	2	2
	Adhesion	0	0	3
	Signal transduction	0	0	2
		48	41	89

Transcript levels and RF values of selected genes were confirmed by quantitative real time PCR (qRT-PCR) (Fig. 2c and Additional file 4).

### Class 1 and Class 2 genes uncover extensive rearrangement of the cell surface in response to HWS

To make the analysis more stringent for genes specifically expressed in response to HWS, we defined Class 1 genes as those that were up-regulated at least two-fold in the mutant (Red) with an RF ≥2 (Table 2). These genes were less induced (Red), unchanged (White) or repressed (Green) in the wild type (relative to T_0_) and are represented in Table 2 by the Red/Red, Red/White or Red/Green ratio respectively. The number of genes was 52, 45 and 40 at 1, 3 and 5 hours of hyphal development, respectively. As shown in Table 2, the “Cell wall” category was among the largest and comprised: (*i*) five cell wall structural mannoproteins (*PGA23*, orf19.750, *RBR1*, *PGA13* and *PGA54*) found only in *C. albicans* or *Candida* spp., (*ii*) three cell wall proteins that have homologs in *S. cerevisiae* (*RBT4, ECM331* and *PGA6*), (*iii*) *TOS1, PLB3* and *EXG2*, enzymes that act on glucans or lipids (*PLB3*), (*iv*) *CRH11*, encoding a glucan cross-linking enzyme of family GH16 and (*v*) *CHS2* and *CHS8*, encoding two of the four isoenzymes of chitin synthase (Chs1p, Chs2p, Chs8p and Chs3p). Moreover, the transcript of *CHS7*, encoding a dedicated chaperone for Chs3p export from the ER (Protein folding/modification category) was also more abundant in the mutant at 5 h.

**Table 2:** Selected Class 1 genes ordered into different biological processes

Functional category	Locus name (ORF n)	*Sc* best hit	1 h		3 h		5 h		Description
			RF	colour ratio	RF	colour ratio	RF	colour ratio	
Cell wall	*PGA23*				22.2	R/G	65.6	R/G	GPI protein
	orf19.750				11.1	R/G	36.0	R/G	In vitro substrate of Kex2p
	*RBR1*						6.7	R/G	GPI protein
	*RBT4*	*PRY3*	2.2	R/R	10	R/G	8.9	R/W	PRY family protein
	*ECM331*	*PST1*	2.3	R/R	5.2	R/W	5.3	R/W	GPI protein
	*PGA6*	*CCW12*	2.8	R/R	6.8	R/W	3.4	R/W	GPI protein
	*CHS8*	*CHS1*	2.2	R/R	2.0	R/R	4.3	R/R	Chitin synthase
	*PGA13*				4.1	R/W	3.7	R/R	GPI protein
	*CRH11*	*CRH1*	4.7	R/R	4.2	R/W	3.3	R/R	GPI transglycosylase
	*PGA54*		2.0	R/R	3.5	R/R			GPI protein
	*TOS1*	*TOS1*			2.5	R/W			Putative β(1,3)-glucanase
	*PLB3*	*PLB1*			2.3	R/W			GPI anchored phospholipase B
	*CHS2*	*CHS1*	2.4	R/R	2.0	R/W			Chitin synthase
	*EXG2*	*EXG2*	2.2	R/R					GPI-anchored exo-β-1,3-glucosidase
Protein folding/ modification	*CHS7*	*CHS7*					2.4	R/W	Export Chs3p from ER
	*CWH8*	*CWH8* ^*a*^					2.0	R/R	Dolychyl-P-P phosphatase of the ER
	*MNT1*	*KTR1*	2.4	R/W					Golgi α(1,2)-mannosyltransferase
	orf19.6864		2.5	R/W					Ubiquitin ligase complex
Filamentous growth	orf19.1208		2.6	R/W			3.1	R/W	*FGR6*-related gene
	orf19.6896		3.4	R/W			2.9	R/W	*FGR6*-related gene
	*FGR6-1*		3.9	R/W	4.3	R/W	2.9	R/W	Member of *FGR* family
	*FGR6*		2.9	R/W	4.6	R/W	2.5	R/W	Member of *FGR* family
	*FGR6-3*		2.5	R/W	2.7	R/W	2.2	R/W	Member of *FGR* family
	*FGR6-10*		3.3	R/W			2.1	R/W	Member of *FGR* family
	*FGR6-4*		2.6	R/W			2.0	R/W	Member of *FGR* family
	orf19.5775		6.7	R/W					*FGR6*-related gene
	orf19.4246	*YKR070W*	2.7	R/W					Tn mutation affects filamentation
Stress response	*DDR48*	*DDR48*			4.5	R/G	3.4	R/W	Immunogenic stress-associated protein
	orf19.2125		2.1	R/R	3.1	R/R	2.4	R/R	CipC-like antibiotic response protein
Signal transduction	*CPP1*	*MSG5*					5.2	R/W	MAPK phosphatase
	*SSK2*	*SSK2*			2.1	R/W	4.7	R/W	MAPKKK regulates Hog1p
	*ARF3*	*ARF3*							Small GTPase
Cell cycle	*PCL1*	*PCL1*					11.7	R/G	Cyclin homolog
	*PCL2*	*PCL2*					6.7	R/G	Cyclin homolog
	*CCN1*	*CLB3*	3.5	R/W	6.0	R/G	7.9	R/W	G_1_ cyclin
	*GIN4*	*GIN4*	2.0	R/R	4.0	R/W	3.2	R/W	Phosphorylates Cdc11p
	*DUN1*	*DUN1*					2.1	R/W	Cell-cycle checkpoint protein kinase
	*CDC28*	*CDC28*			2.0	R/W	2.4	R/R	Cyclin-dependent kinase
	*HSL1*	*HSL1*	2.9	R/W	2.2	R/W			Morphogenesis regulation
	*INT1*	*BUD4*			2.1	R/W			Morphogenesis regulation
Cell polarity/ cytoskeleton	*MLC1*	*MLC1*	2.0	R/R					Cytokinetic ring in hyphae
	orf19.3501	*PXL1*			2.1	R/W			Polarized growth
	orf19.6610	*STU2*					2.9	R/W	Microtubule-associated protein
DNA replication/repair	*POL30*	*POL30*	2.6	R/W			2.6	R/W	PCNA
	*POL3*	*POL3*					2.2	R/W	Catalytic subunit of DNA pol delta
	*RAD51*	*RAD51*			3.3	R/W			Homologous recombination and repair
	*DUT1*	*DUT1*	2.3	R/R	3.2	R/W			dTTP *de novo* biosynthesis
	orf19.7425	*UNG1*	2.0	R/R					Uracil-*N*-glycosylase
	*MSH6*	*MSH6*			3.1	R/W			Mismatch repair
	*RFA2*	*RFA2*			2.4	R/W			Putative DNA replication factor A
	*RFA1*	*RFA1*	2.2	R/W	2.1	R/W			Putative DNA replication factor A
	*RNR1*	*RNR1*	2.0	R/R					Subunit of ribonucleotide reductase
	orf19.2796	*POL12*	3.5	R/W					DNA Pol α/primase complex
	*POL1*	*POL1*	3.2	R/W					DNA Pol α
	*CDC54*	*MCM4*	2.4	R/W					Pre-replication helicase complex
	*CDC46*	*MCM5*	2.6	R/W					MCM complex subunit
	*MCM6*	*MCM6*	2.0	R/W					MCM complex component
Metabolism	*GFA1*	*GFA1*	2.8	R/R	2.1	R/R	2.3	R/R	Glucosamine-6P synthase
	*FAS1*	*FAS1*	2.9	R/W	2.0	R/R			β-subunit of fatty acid synthase
	*ACC1*	*ACC1*	2.6	R/W	2.0	R/R			Acetyl-coenzyme-A carboxylase
	*FAS2*	*FAS2*	2.5	R/W	2.0	R/R			α-subunit of fatty acid synthase
	*DPP3*	*DPP1*	3.1	R/R					DGPP phosphatase; farnesol synthesis
	*GNA1*	*GNA1*	2.1	R/R					Glucosamine-6P acetyltransferase
	*RTA2*	*RSB1*	4.8	R/G					Flippase for sphingolipid release
	orf19.2761	*GPI11*	2.3	R/R					Putative GPI anchor assembly protein
Oxido-reduction processes	*PUT1*	*PUT1*	2.3	R/W					Proline oxidase
	orf19.1340	*YDL124W*			3.1	R/R			Member of aldo-keto reductase family
	*GDH3*	*GDH3*			2.5	R/R			NADP-glutamate dehydrogenase
	orf19.2244	*YJR096W*					3.6	R/W	Member of aldo-keto reductase family
	orf19.7306	*YPR127W*					2.0	R/R	Aldo-keto reductase
Chromatin/chromosome	*HHF22*	*HHF2*	2.2	R/R	2.6	R/W			Putative histone H4
	orf19.1052	*HTB1*			3.2	R/W			Putative histone H2B
	*HHF1*	*HHF1*			3.2	R/W			Putative histone H4
	*HTA1*	*HTA1*	2.1	R/R	2.9	R/W			Histone H2A
	*HTA2*	*HTA1*			2.4	R/W			Putative histone H2A
	*HTB1*	*HTB1*			2.2	R/W			Histone H2B
	*IRR1*	*IRR1*	2.3	R/W					Putative cohesin subunit
	*ASF1*	*ASF1*	2.2	R/W					Nucleosome assembly factor
Pathogenesis	*SAP6*	*BAR1*					2.7	R/R	Secreted aspartyl protease
Transcription	*SWI6*	*SWI6*	2.4	R/W					Regulator of G1/S transition
	*YOX1*	*YOX1*	2.2	R/W	2.3	R/R	3.8	R/R	Homeobox transcriptional repressor
	*WOR2*	*UME6*			2.3	R/W	5.1	R/W	Zn2Cys6 regulator of W-O switching
Transport	*CCC2*	*CCC2*					4.2	R/W	Golgi Copper transporter
	*FRP3*	*ATO2*	2.5	R/W					Ammonium transporter
	*FLC2*	*FLC2*	2.5	R/W	2.5	R/W	2.2	R/W	FAD (or putative calcium) transporter
	*YVC1*	*YVC1*	2.0	R/R	2.8	R/W	2.4	R/W	Calcium activated cation channel
	orf19.5022	*SMF2*	2.0	R/W					Divalent metal transporter

The data refer to Class 1 genes showing statistically significant RF (see Additional file 2 and criteria used as described in Methods). Only genes that were upregulated respect to time zero by two-fold in the mutant (expression ratio ≥ 2) are shown. The genes were further colour-labelled according to their expression pattern: R/G (Red/Green, induced in the mutant and repressed in the wild type), R/W (Red/White, induced in the mutant and unchanged in the wild type), R/R (Red/Red, induced in both strains but more in the mutant). Only genes with known function or participation to a biological process are listed. ^a^This gene has two names: *CWH8* and *CAX4*

In addition, *CWH8*, encoding an ER-localized enzyme required for protein *N*-glycosylation (“Protein folding/modification”) and *FLC2* (“Transport”) encoding a putative calcium transport, were two interesting genes with a Red/White pattern. In particular, *FLC2* (*FL*avin *C*arrier 2) was present at all the time points strongly suggesting that its function could be important for adaptation to HWS. The Flc proteins were initially proposed to be putative carriers for FAD entry into the ER [36] but recent findings suggest that they are involved in calcium signaling and in hypotonic shock response [37]. Other functional categories included “Filamentous growth” with the presence of several *FGR* genes, “Stress response” (*DDR48*), “Cell cycle”, “DNA replication and repair” and “Chromatin and Chromosome”. Notably, the genes induced in the mutant but repressed in the wild type, (R/G), included the “Cell wall” genes *PGA23*, orf19.750, *RBR1, RBT4,* the “stress response” gene *DDR48,* the “Metabolism” gene *RTA2,* and the G_1_ cyclins *PCL1, PCL2* and *CCN1*, which points to a core HWS response that primarily affects cell wall and cell cycle progression.

Table 3 reports genes repressed during HWS (Class 2). Only genes down-regulated at least 2-fold in the mutant (Green) with an RF ≤ 0.5 [induced in the wild type (Red), unchanged in the wild type (White) or repressed in the wild type, but less than in the mutant (Green)] were selected. The reduced transcript levels of three “Adhesion” genes accord with the adhesion defects previously described for the mutant [31]. In agreement with other reports, the category “RNA processing and biogenesis” and “Metabolism” were particularly rich [38, 39]. Whereas “Metabolism” genes were more affected at 5 h, “RNA processing and biogenesis” genes were more affected at 1 h. The “Transcription” category contained a number of transcription factors and notably *CRZ2*, a paralog of *CRZ1*, was more repressed in the mutant than in the wild type.

**Table 3:** Selected Class 2 genes ordered into different biological processes

Functional category	Locus name (ORF n)	*Sc* best hit	1 h		3 h		5 h		Description
			RF	colour ratio	RF	colour ratio	RF	colour ratio	
Adhesion	*ALS2* orf19.1097	*SAG1*					0.2	G/G	GPI protein; Adhesin
	*ALS2* orf19.2121	*SAG1*					0.2	G/W	GPI protein; Adhesin
	*ALS4*	*SAG1*					0.4	G/G	GPI protein; Adhesin
	*PGA38*		0.4	G/W					Adhesin-like GPI protein
Cell wall	*SIM1*	*UTH1*			0.3	G/G			SUN family member
Metabolism	*GAL1*	*GAL1*					0.2	G/R	Galactokinase
	*GAL7*	*GAL7*					0.3	G/W	Galactose-1-P- uridyl transferase
	*GAL10*	*GAL10*					0.3	G/W	UDP-glucose 4-epimerase
	*YHB5*	*YHB1*			0.2	G/G	0.2	G/G	Detoxifying Flavohemoglobin
	*YHB1* ^*a*^	*YHB1*					0.1	G/G	Nitric oxide dioxygenase
	*AAH1*	*AAH1*			0.5	G/G			Adenine deaminase
	*ARO3*	*ARO3*					0.5	G/W	Aromatic amino acid synthesis
	*ARO10*	*ARO10*	0.4	G/G					Aromatic decarboxylase
	*ICL1*	*ICL1*	0.5	G/G					Isocitrate lyase
	*IFE2*	*BDH1*					0.1	G/G	Alcohol dehydrogenase
	orf19.7593	*ASP1*					0.5	G/G	Asparaginase
	*FEN1*	*ELO2*					0.5	G/G	Fatty acid elongase
	orf19.3483	*PGC1*					0.5	G/G	Phosphatidyl Glycerol phospholipase C
	*CDG1*						0.2	G/W	Cysteine dioxygenase
Oxido-reduction processes	*FRE7*	*FRE3*	0.4	G/W					Copper regulated cupric reductase
	*COX15*	*COX15*					0.5	G/G	Cytochrome oxidase assembly protein
	orf19.5394.1	*PET191*					0.5	G/W	Cytochrome c assembly
	*ABC1* ^a^	*COQ8*					0.5	G/W	Ubiquinol Cytochrome c reductase
Protein folding/modification	orf19.3301	*MET30*	0.4	G/G	0.5	G/G	0.4	G/G	Part of ubiquitin ligase complex
	*ERO1*	*ERO1*			0.5	G/G	0.4	G/G	Oxidative protein folding in the ER
	*MNN1*	*MNN1*	0.4	G/G			0.4	G/G	Putative α(1,3)-mannosyltransferase
RNA processing and biogenesis	*ENP1*	*ENP1*	0.4	G/W					Pre-rRNA processing
	*RMS1*	*RKM4*	0.5	G/W					Ribosomal lys methyltransferase
	orf19.2314	*CGR1*	0.5	G/W					Nucleolar integrity and r-RNA processing
	*PRP5*	*PRP5*	0.5	G/W					RNA helicase
	*UTP18*	*UTP18*	0.5	G/W					Maturation rRNA
	orf19.494	*NAF1*	0.4	G/G	0.5	G/G	0.5	G/G	RNA-binding protein
	orf19.3303	*PPM2*	0.5	G/G	0.5	G/G			tRNA methyltransferase
	orf19.2934	*BUD20*	0.4	G/G					C2-H2 Zinc finger protein required for ribosome assembly
	orf19.6234	*IPI3*	0.4	G/G					Putative U2 snRNP component
	*JIP5*	*JIP5*	0.4	G/G					Biogenesis of Large ribosome subunit
	*MAK16*	*MAK16*	0.4	G/G					Constituent 66 S pre ribosomal particle
	orf19.154	*UTP30*	0.5	G/G					U3-containing protein
	orf19.1642	*LOC1*	0.5	G/G					Localization mRNA
	*NSA1*	*NSA1*	0.5	G/G					Large ribosome subunit biogenesis
	orf19.809	*NOP12*	0.5	G/G					Maturation rRNA precursor
	*NOG2*	*NOG2*	0.5	G/G					Associates with 60 S subunit
	*RRP8*	*RRP8*	0.5	G/G					Ribosomal protein
	*CHR1*	*ROK1*	0.5	G/G					RNA helicase pre rRNA processing
	*RRP9*	*RRP9*	0.5	G/G					Ribosomal protein
	orf19.3479	*PSU1*					0.5	G/G	tRNA:pseudouridine synthase
	*TRM12*	*TRM12*					0.5	G/W	tRNA methyltransferase
	*SEN2*	*SEN2*					0.5	G/G	tRNA splicing endonuclease
	orf19.6736	*GEP3*					0.5	G/G	Mitochondrial ribosome biogenesis
Signal transduction	*PPT1*	*PPT1*					0.5	G/W	Ser/Thr protein phosphatase
Stress response	*HSP21*				0.3	G/G	0.1	G/W	Small heat-shock protein
	*HSP78*	*HSP78*			0.4	G/W	0.5	G/G	Heat-shock protein
Transcription	*ZCF3*		0.3	G/W	0.4	G/W	0.4	G/W	TF for filamentation
	*TRY6*		0.5	G/G			0.3	G/G	H-L-H TF for adherence
	*CRZ2*	*CRZ1*	0.4	G/G			0.4	G/G	C2H2 TF repressed at pH 8
	*CTA4*	*OAF1*			0.5	G/G			Zn2Cys6 TF
	*GLN3*	*GLN3*			0.5	G/W			GATA TF
	*TYE7*	*TYE7*					0.2	G/W	HLH TF, glycolysis, CS-repressed
	*CUP9*	*TOS8*					0.3	G/W	Transcription factor
	*ARG83*	*ARG81*					0.5	G/W	Gal4p-like TF
	*CPH2*	*HMS1*					0.5	G/W	Myc-bHLH TF promotes Hyphal growth
	*FCR1*	*CAT8*					0.3	G/G	Zn cluster TF, fluconazole resistance
Transport	orf19.6578		0.5	G/W					Membrane transported CS repressed
	*SSU1*	*SSU1*	0.4	G/G	0.3	G/G	0.3	G/G	Sulfite transport protein
	*PHO89*	*PHO89*	0.5	G/G			0.5	G/G	Phosphate permease
	*QDR1*	*QDR1*			0.5	G/G	0.2	G/G	Multidrug transporter CS repressed
	*GIT2*	*GIT1*			0.5	G/G	0.5	G/G	Glycerolphosphoinositol permease
	*TPO4*	*TPO4*			0.5	G/W			Spermidine transporter
	*PTR2*	*PTR2*					0.4	G/G	Peptide transporter, repressed pH 8
	*HGT6*	*HXT6*					0.3	G/W	Glucose transporter
	*DIP5*	*DIP5*					0.5	G/W	Dicarboxylic amino acid permease

The data refer to Class 2 genes showing statistically significant RF (see Additional file 3 and criteria used as described in Methods). Among the genes present in Additional file 3, only genes that were downregulated by two-fold in the mutant (expression ratio ≤ 0.5 with respect to time zero) are shown. The genes were further labelled by a colour ratio according to their induction pattern in the mutant with respect to the wild type: G/R (Green/Red, repressed in the mutant and induced in the wild type), G/W (Green/White, repressed in the mutant and unchanged in the wild type), G/G (Green/Green, repressed in both strains but more in the mutant). Only genes with known function or participation to a biological process are listed. ^a^nomenclature conflict; TF: transcription factor

### Genetic interactions of *PHR1* and *CHSx* genes during hyphal development

Transcriptional data (Table 2) suggested that the absence of *PHR1* forces multiple adjustments in the cell’s chitin synthesis: *CHS8*, *CHS2* and *CHS7* have RF >2. Despite it being well known that chitin plays an important compensatory role in protecting damaged fungal wall from lysis, studies in *C. albicans* have been primarily performed in unbuffered YPD. Here, we analyzed the role of chitin synthases in the HWS response. *CHS1* is an essential gene required for septum formation and for cell wall integrity. *CHS2* is preferentially expressed in hyphae where it contributes to overall chitin synthesis as well as septum formation. *CHS8* is responsible for the synthesis of a particular type of long fibrillar chitin at the septum [40–44]. *CHS3* contributes the majority of cell wall chitin, which is deposited in the septal ring and lateral walls, of yeast and hyphae [45, 46]. Moreover, *CHS3* is responsible for Calcofluor white (CW)/CaCl_2_ induced chitin accumulation which protects *C. albicans* from the lethal effect of echinocandin [47]. *CHS2, CHS8* and *CHS3* are also responsible for the remedial septum that is synthesized when Chs1p is inhibited, an indication of cross compensation [40].

**Fig. 3:**
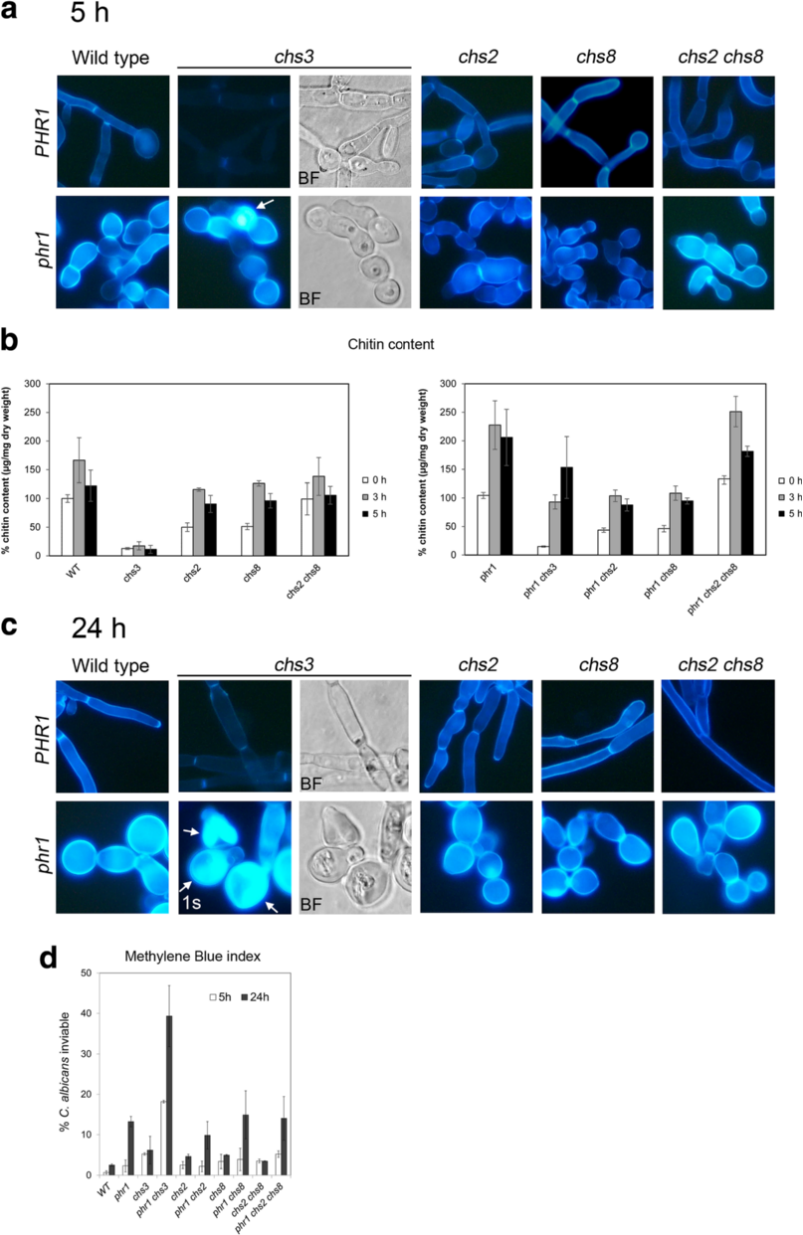
Effects of deletion of *CHS3, CHS2*, *CHS8* or *CHS2* and *CHS8* on the *PHR1* null mutant phenotype. **a** *Upper panel*: strains CAI4 (wild type), Myco3 (*chs3Δ*), C155 (*chs2Δ*), NGY128 (*chs8Δ*) and NGY138 (*chs2Δ chs8Δ*) and *in the lower panel* their respective *phr1*Δ derivatives CAS8 (*phr1*Δ), FP3 (*phr1*Δ *chs3*Δ), FP155 (*phr1*Δ *chs2*Δ), FP128 (*phr1*Δ *chs8*Δ) and FP138 (*phr1*Δ *chs2*Δ *chs8*Δ). Blastospores were induced to switch to hyphal growth in M199-150 mM HEPES-pH 7.5 at 37 °C and 5 hours later aliquots of culture were processed for CW-staining without fixation. Pictures were taken using the same exposure time (2.5 s). For *chs3*Δ and *phr1*Δ *chs3*Δ the corresponding bright field (BF) image is also shown. The arrow indicates a cell in which CW penetrated. **b** Chitin content at time zero, 3 and 5 hours. The mean value of each strain is expressed as a percentage of the wild type value at time zero [100 %,12.50 μg of GlcNac/mg d.w. (dry weight of cells) ± 0.84 standard deviation (SD)] (*n* = 3). **c** Same as in **a** but cells were collected 24 hours after induction of hyphal growth. Due to permeability of CW into the cells, the image exposure time for *phr1*Δ *chs3*Δ cells was reduced to 1 s. The corresponding BF image shows cell ghosts of highly fluorescent cells. Magnification, x 1,300. **d** Determination of the percentage of dead cells by MB staining in M199-pH 7.5. Data are mean values ± S.D. For the parental strains a single category “dead hyphae” is shown and includes: dead mother cells, hyphae with dead apex or with dead mid-compartments or long dead hyphae. At least 500 hyphae were counted in triplicate and percentage of “dead hyphae” are shown. For the *phr1*Δ mutants, samples were sonicated for 5 s before MB staining. Blue cells were counted over a total of at least 500 cells

A *PHR1* homozygous null mutation was introduced into strains lacking *CHS2*, *CHS3, CHS8* and both *CHS2* and *CHS8* and analyzed during the induction of hyphal growth. At 5 hours after the shift, all *chsx*Δ mutants had formed hyphae but they were altered in morphology and were highly flocculant (Fig. 3a). Hyphae of *chs2*Δ and *chs8*Δ mutants were larger in diameter and hyper-branched. These traits were accentuated in the double mutant *chs2*Δ *chs8*Δ cells. CW-staining, specific for chitin, was comparable to the wild type strain except for *chs3*Δ cells that were CW-positive only at the thin line of the septa, an observation in agreement with Chs3p being responsible for the deposition of the majority of chitin located in the lateral wall and chitin ring but not in the chitin disk of the primary septum [48, 49]. As shown in Fig. 3a (*lower panel*), CW-fluorescence of the *phr1*Δ mutant was distinctly more intense than in the control strain distributed over the wall and septa. Deletion of *PHR1* conferred the same morphological defects to the *chsx*Δ mutants as seen with *PHR1* deletion alone but cells were more aggregated and *phr1*Δ *chs3*Δ cells arrested earlier. Unexpectedly, the *phr1*Δ *chs3*Δ mutant stained with similar intensity and distribution as the *phr1*Δ mutant and also showed cells in which the dye had penetrated inside, an index of increased susceptibility to lysis, suggesting cell lysis was occurring (Fig. 3a, *arrow*). Conversely, combined deletion of *PHR1* and either *CHS2* or *CHS8* resulted in reduced CW-staining, whereas the simultaneous absence of both enhanced CW staining.

Additionally, we measured the chitin content of the cells. As shown in Fig. 3b, the deletion of *CHS3* in a wild type background dramatically reduced the chitin content of the cells and remained largely unchanged during the induction of hyphal growth. Loss of either *CHS2* or *CHS8*, alone or in combination, resulted in a small reduction in chitin content compared to the wild type. In contrast, the chitin increase observed in the *phr1*Δ mutant was reduced in the absence of *CHS3* or either of *CHS2* or *CHS8.* Unexpectedly, an increased chitin content was restored in the *phr1*Δ *chs2*Δ c*hs8*Δ suggesting that the combined loss of *CHS2* and *CHS8* generates further stress that is compensated by the other isoenzymes. In addition, the chitin content of *phr1*Δ *chs3*Δ cells progressively increased during blastospore germination despite the aberrant appearance of the cells.

Microscopic examination of the *phr1*Δ *chs3*Δ mutant after 24 h incubation revealed swollen, lysed cells and ghosts of cells into which CW had penetrated (Fig. 3c, *arrows*). This prompted an examination of the viability of the various mutants using MB staining. A weak lysis defect for the hyphae of the parental strains was detected especially for the *chs3Δ* mutant (Fig. 3d). Only a few percent of *phr1*Δ cells were inviable at 5 h and this increased to around 15 % at 24 h, whereas 20 % of the *phr1*Δ *chs3*Δ cells were dead within 5 h and this increased to about 40 % by 24 h (Fig. 3d). When the *phr1*Δ mutation was combined with deletions of *CHS2* and/or *CHS8* no relevant effect on viability was observed after 24 h (Fig. 3d). At pH 8, the percentage of dead *phr1*Δ *chs3*Δ cells increased to 70 % whereas the other strains remain similar to *phr1*Δ (about 20 %). In conclusion, Chs3p is required for a sustained HWS response in liquid M199-pH 7.5 at 37 °C.

Interestingly, Chs2p and Chs8p appear to contribute to the synthesis of chitin during the initial 5 hours of hyphal development. However, increased chitin content *per se* does not provide cell wall integrity in the absence of Phr1p. The *phr1*Δ *chs3*Δ mutant accumulates chitin but partially fails to maintain cell integrity.

**Fig. 4:**
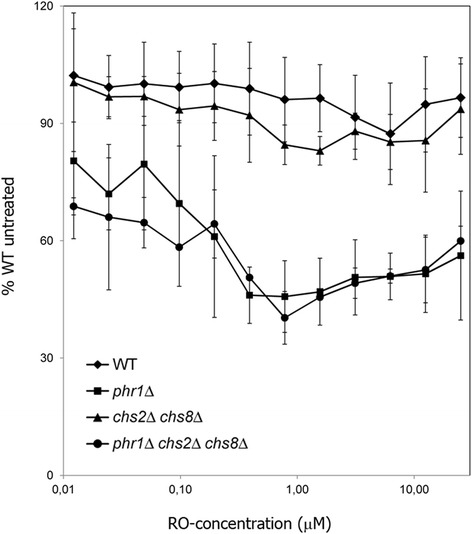
Relative increased susceptibility to Chs1p inhibition of *phr1*Δ and *phr1*Δ *chs2*Δ *chs8*Δ mutants. The mutants CAS8 (*phr1*Δ) and FP138 (*phr1*Δ *chs2*Δ *chs8*Δ) and their parental strains CAI4 (wild type) and NGY138 (*chs2*Δ *chs8*Δ) were analyzed for sensitivity to RO-09-3143 by microdilution assay in M199-pH 7.5 37 °C, at an inoculum size of 5 x 10^5^/ml. Data refer to 24 h. The results derive from 3 biological replicates and 2 microtiter plates for each replicate. Data are relative to untreated wild type which was set to 100 % (mean A_595_ = 0.600 ± 0.063 S.D.). The MB staining of cells from a representative experiment is shown in Additional file 5

To assess which isoenzyme/s contributes to chitin synthesis when *CHS3, CHS2* and *CHS8* are missing, we tested by microdilution assay the sensitivity of the strains to RO-09-3143, a specific inhibitor of Chs1p at inoculum size of 5 x 10^5^ cells/ml [50]. The absence of visible growth of the *phr1*Δ *chs3*Δ mutant precluded testing the inhibitor against this strain. Interestingly, *phr1*Δ and *phr1*Δ *chs2*Δ *chs8*Δ mutants exhibit similar sensitivity to the drug but both were more susceptible than the parental strains. This result indicates that Chs1p contributes to the growth of both mutants (Fig. 4). We also examined the lysis phenotype by MB staining at two different drug concentrations. *phr1*Δ and *phr1*Δ *chs2*Δ *chs8*Δ mutants were more prone to lysis compared to their parental strains (Additional file 5). At the highest concentrations of inhibitor all strains were mostly lysed in accord with the essential role of Chs1p. These results suggest that Chs1p is required for growth in *phr1*Δ cells but the additional absence of *CHS2* and *CHS8* does not further affect the phenotype.

### Chs3p and Chs8p are essential for germination and growth of *phr1*Δ cells on solid filamentation media

Because some mutations differentially influence filamentation in broth culture versus solid media, we also tested the phenotype of the mutants on agar solidified media using the nutrient-rich M199 medium and an alternative medium (Spider). In both conditions, the parental strains formed filaments whereas the mutants containing a *phr1*Δ mutation were unable to filament, as observed in broth culture.

**Fig. 5:**
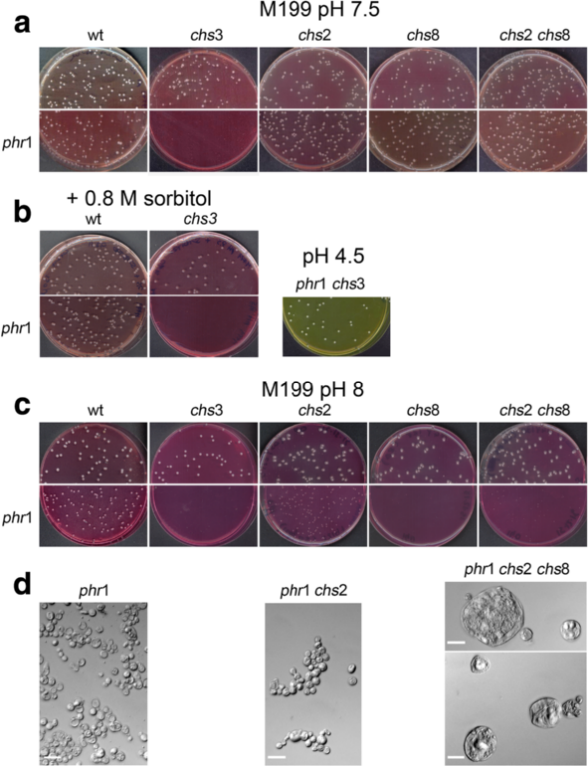
Genetic interactions of *CHS3, CHS2, CHS8*, *CHS2/CHS8* and *PHR1* during filamentation on solid M199 at pH 7.5 and pH 8. Blastospores were plated as single cells on solid M199-150 mM HEPES buffered at the indicated pH supplemented with uridine 100 μg/ml, and incubated at 37 °C (*n* = 4). **a** Colonies produced after 24 hours of incubation at pH 7.5 **b** Inclusion of sorbitol as an osmotic stabilizer does not suppress lethality of *phr1Δ chs3Δ* (*left panel*) whereas the double mutant exhibits normal growth at pH 4.5, a permissive pH (*n* = 4). **c** Colonies produced on solid M199-150 mM HEPES pH 8 incubated for 3 days at 37 °C (*n* = 4). **d** Microscopic morphology of cells taken from the indicated colonies on plate at pH 8. The scale bar is 20 μm

After 24 hours on solid M199-pH 7.5 at 37 °C, all the strains gave rise to visible colonies except the *phr1*Δ *chs3*Δ mutant (Fig. 5a). Upon microscopic observation of the agar surface, we concluded that *phr1*Δ *chs3*Δ cells did not germinate indicating that cell death is an early event. Inclusion of 0.8 M sorbitol in the plates did not suppress the lethal phenotype (Fig. 5b). The inability to germinate of the *phr1*Δ *chs3*Δ mutant was suppressed at pH 4.5 confirming the association of the synthetic lethal phenotype with the presence of the *PHR1* deletion (Fig. 5b).

On M199-pH 8, germination was delayed relative to pH 7.5, for the parental strains and further delayed in mutants harboring the *phr1*Δ mutation, in agreement with the more restrictive pH. After 3 days, as expected, *phr1*Δ *chs3*Δ cells did not grow, but surprisingly the *phr1*Δ *chs8*Δ mutant also failed to produce visible colonies (Fig. 5c). In contrast, *phr1*Δ *chs*2Δ mutants did form small colonies. The triple *phr1*Δ *chs*2Δ *chs8*Δ mutant gave rise to hardly visible microcolonies and cells taken from these colonies were swollen round and abnormally big, indicating a more severe morphological phenotype compared to *phr1*Δ or *phr1*Δ *chs2*Δ (Fig. 5d). Upon microscopic observation of the agar surface, *phr1*Δ *chs8*Δ colonies appeared as small aborted colonies indicating that they germinated but died before producing a visible colony (Additional file 6, *arrows*). The phenotype of the *phr1*Δ *chs8*Δ cells and of the *phr1*Δ *chs*2Δ *chs8*Δ strain was not sorbitol-remediable (data not shown). Therefore on M199, *CHS3* is essential for germination of *phr1*Δ cells at restrictive pH. *CHS8* is essential both alone or combined with *CHS2*, for maintaining growth and viability of the germinated *phr1*Δ cells, but only at pH 8.

**Fig. 6:**
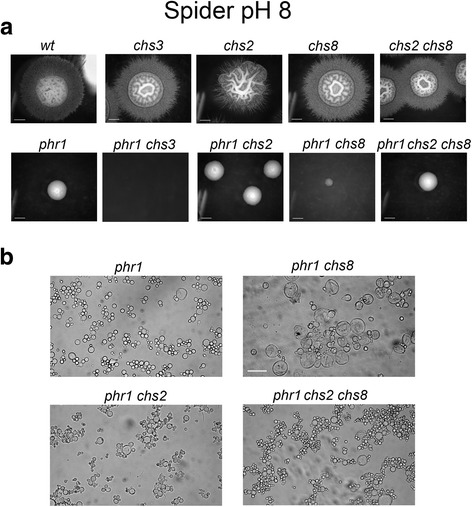
Genetic interactions of *CHS3, CHS2, CHS8*, *CHS2/CHS8* and *PHR1* during filamentation on Spider pH 8 at 37 °C. Blastospores were plated as single cells on solid Spider-150 mM HEPES buffered at pH 8 (*n* = 3). **a** Colonies photographed after 7 days of growth at 37 ° C. The scale bar is 2 mm. **b** Microscopic morphology of cells taken from the colonies. The scale bar is 40 μm

Since solid Spider medium has a pH around 6, it was supplemented with 150 mM HEPES and buffered at the desired pH in order to test the phenotype of the mutants. After 7 days on Spider-pH 7.5 at 37 °C, all the strains generated colonies (Additional file 7). At pH 8, the *phr1*Δ mutant gave rise to small colonies, whereas the *phr1*Δ *chs3*Δ mutant did not produce colonies. This lethal phenotype was not sorbitol-remediable (Fig. 6a and Additional file 7). Moreover, the *phr1*Δ *chs8*Δ double mutant produced either no or less than 5 visible colonies per plate (*n* = 3). The inclusion of sorbitol in the plates partially meliorated the phenotype as more small colonies were produced (data not shown). Microscopic examination of cells taken from the colonies on Spider-pH 8 evidenced the presence of lysed cells and cell ghosts in the few visible colonies of *phr1*Δ *chs8*Δ, suggesting that extensive lysis occurred after germination (Fig. 6b). In contrast, the morphology of *phr1*Δ *chs2*Δ and *phr1*Δ *chs2*Δ *chs8*Δ cells was similar to *phr1*Δ cells except that *chs*Δ derived cells were more aggregated (Fig. 6b). Thus, a partial attenuation of the phenotype occurred when both *CHS8* and *CHS2* were absent.

In conclusion, the phenotype of *phr1*Δ *chsx*Δ mutants in solid medium is influenced by the composition of the medium and by pH, which affects the severity of the stress. The results indicate a unique and essential role of *CHS3* and *CHS8* for *phr1*Δ viability in solid media (see further Discussion).

### The block of hyphae elongation up-regulates DNA replication genes and accelerates progression into the DNA division cycle

**Fig. 7:**
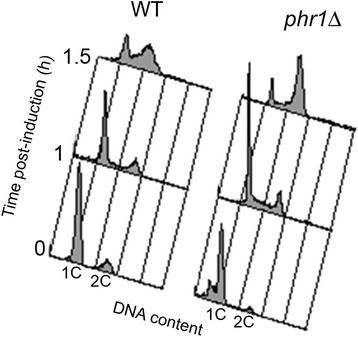
Cytofluorimetric analysis of DNA distribution. Cells were analyzed after induction of hyphal growth at 37 °C in M199 buffered at pH 7.5. 1C and 2C indicate the relative DNA content

Next, we investigated the Class 1 genes related to “DNA replication and repair” (*POL30, POL3, RAD51, DUT1, MSH6, RFA2, RFA1,* orf19.7425*,* orf19.2796*, POL1, CDC54, RNR1, EXO1, orf19.4030, CDC46, MCM6*) and “Chromatin and chromosomes” (*HHF22, HHF1, HTA1*, *HTA2, HTB1, IRR1, ASF1, SMC6, NAT4,* orf19.1052) (Table 2 and Additional file 2). This transcriptional response occurred at 1 and 3 h and may be related to the block of hyphal elongation. However, the actual effect of these changes on entry into S-phase has not been reported. Therefore, we analyzed by flow cytometry the DNA distribution profile of wild type and *phr1*Δ mutant strains undergoing the Y-H transition. Blastospores after prolonged incubation in stationary phase (1.5 days) showed a very high degree of synchrony in germ tube formation when inoculated into hyphal induction conditions (M199-pH 7.5). The percentage of germ tubes was about 88 % for both the wild type and *phr1*Δ mutant at 1 h. By 1.5 h, thin hyphae already appeared in the wild type whereas the germ tubes of the mutant developed a swollen apex. At this time, *phr1*Δ cells have already entered S-phase, earlier than control cells, and also an enrichment of cells at G_2_/M was observed (Fig. 7). These results are in agreement with the early induction of DNA replication genes in the mutant.

## Discussion

The present study describes the impact of the loss of β(1,3)-glucan assembly on the transcriptional program of *C. albicans* cells undergoing hyphal development. Cells lacking the β(1,3)-glucan transferase activity of Phr1p were also the object of further genetic and functional studies to test the physiological relevance of the observed increase of *CHS2* and *CHS8* transcript levels. To our knowledge this is the first report in which the stress generated by defective hyphal wall assembly was analyzed by a large-scale approach and the stress was not imposed by use of inhibitors or temperature.

### Glucan remodeling and hyphal wall integrity

First, our results indicate that cells deficient in β(1,3)-glucan remodeling activate the hyphal transcriptional program as shown not only by the ability to initiate germ tube formation but also by the induction of typical hyphal-induced genes. Secondly, none of the *PHR* genes (*PHR2*, *PHR3*, *PGA4* and *PGA5*) were identified in our analysis indicating that no transcriptionally-mediated cross compensatory mechanism exists between these paralogs and *PHR1*. The hyphal wall defects triggered a complex HWS response whose main elements are here summarized.

### A core set of eight mannoproteins responds to the loss of β(1,3)-glucan assembly

Among the genes encoding cell wall proteins that were up-regulated in the *phr1*Δ mutant we identified five *Candida-*specific cell wall structural mannoproteins (*PGA23*, orf19.750, *RBR1*, *PGA13* and *PGA54*). We included the uncharacterized orf19.750 since the predicted polypeptide has features in common with surface mannoproteins: the presence of a signal peptide, abundance of alanine, serine and threonine residues, a hydrophobicity plot similar to that of Ece1p and the presence of internal repeats. Furthermore, the recombinant protein was also shown to be a substrate in vitro of the Kex2 protease [51]. Three other cell wall proteins (*RBT4, ECM331* and *PGA6*) have homologs in *S. cerevisiae* and have been partially characterized [52, 53]. *PGA6* is the ortholog of *CCW12*, a *S. cerevisiae* gene encoding a covalently-linked cell wall mannoprotein required for cell wall integrity [54]. Interestingly, *PGA13* encodes a mannoprotein that influences surface properties and its deletion increases adhesion of cells to plastic [53]. We postulate that consequently enhanced transcription of *PGA13* in the *phr1*Δ mutant may result in reduced adhesion. *PGA13* up-regulation, combined with the observed down-regulation of *ALS2, ALS4* and *PGA38* (encoding an adhesin-like protein) may explain the reduced adhesion to plastic and cell monolayers we previously described for *phr1*Δ cells [31]. As mannoproteins are an abundant constituent of the cell wall, altogether this response suggests a revamping and the reinforcement of the extracellular matrix during HWS.

The increase of *CWH8* transcript level at 5 h is intriguing (Table 2). In *S. cerevisiae,* the dolichyl pyrophosphate (Dol-P-P) phosphatase of the ER encoded by *CWH8* converts DolP-P to Dol-P (dolichyl monophosphate) in the luminal leaflet of the ER and Dol-P is reutilized for the synthesis of the lipid-linked core-oligosaccharide chain [55]. The Cwh8p-catalyzed reaction represents a limiting step in the rate of protein glycosylation and the increase of the *CWH8* transcript seems consistent with the need of active mannoprotein synthesis in the *phr1*Δ mutant.

### Crh11p transglycosidase and the HWS response

Among the “Cell wall” genes, the *CRH11* transcript was more abundant in the *phr1*Δ mutant at all time points. *CRH11* is the only member of a family of three genes that includes *UTR2/CSF4* and *CRH12,* to respond to cell wall stress at the transcriptional level [56]. Its homolog *ScCRH1* is also up-regulated in cell wall mutants and in cells treated with Congo red or zymolyase [57, 58]. *Sc*Crh1p acts in vitro and in vivo in the cross-linking of chitin chains to β(1,3)-and β(1,6)-glucan which in turn can be linked to GPI-mannoproteins yielding a more resistant wall [59, 60]. Assuming a functionally analogous role, *C. albicans* Crh11p combined with the observed increase in chitin and mannoprotein expression, could enhance the establishment of new cross-linkages between chitin-glucan and GPI-mannoproteins to protect cells from lysis. Thus, *CRH11* is at the core of both the cell wall and hyphal wall stress response.

### Comparison with the Caspofungin-induced cell wall stress response

Caspofungin (CS) induces cell wall stress by inhibiting β(1,3)-glucan synthesis and the response to this cellular insult might be expected to partially overlap the response to loss of Phr1p. Bruno et al. (2006) defined a core set of 34 CS-responsive genes by combining their results and those of Liu et al. [23, 61]. Of the *PHR1*-responsive genes, 9 were in common with the core CS-responsive genes defined after 1 h-treatment: four were down-regulated (orf19.5267, orf19.6578, *TYE7* and *SSU1*) whereas five were up-regulated (*CRH11*, *PGA13*, *ECM331*, *DDR48* and orf19.3615). Three of these genes (*CRH11*, *PGA13*, *DDR48*) showed sustained expression in the *phr1*Δ mutant whereas the others declined at 3 h (Table 2 and Additional file 2), suggesting these genes may be more critical to the cell’s attempt to counteract hyphal wall damage. In addition, *PGA23*, also a CS-induced gene [61], is persistently up-regulated in the *phr1*Δ mutant. Notably, the expression of *CRH11*, *PGA13, PGA23, ECM331* is governed by *CAS5*, encoding a zinc-finger transcription factor that plays a major role in cell wall damage response [61]. In our study, *CAS5* is repressed in the wild type but not in the *phr1*Δ mutant (Additional file 2).

Lastly, *CHS2* and *CHS8* are up-regulated in CS-treated cells [47] as in *phr1*Δ mutant. The MAP kinases Mkc1p and Hog1p, and also Ca^++^-calcineurin were demonstrated to contribute to *CHS2* and *CHS8* up-regulation [47].

### *CHS3* and *CHS8* preserve *phr1*Δ cell integrity in different conditions of filamentation

Among the four chitin synthases of *C. albicans*, the role of *CHS2* and *CHS8* in cell wall stress is still poorly understood. Their transcript levels increase not only in response to CS, but also following treatments with CaCl_2_ and CW, which stimulates chitin synthesis [35, 47]_._ While this correlates with an increase in the in vitro activity of these enzymes, there is no corresponding increase in chitin accumulation in vivo [47]. Our functional analyses demonstrate that *CHS3* is crucial during hyphal growth in the absence of Phr1p and support the notion that Chs3p is the major compensatory chitin synthase both in cell wall stress and HWS in fungi [21, 28, 47, 62, 63]. However, our results also provide new hints to the understanding of the biological role of Chs2p and Chs8p in the response to HWS. Interestingly, Chs2p and Chs8p contribute to the chitin increase in the *phr1*Δ mutant during filamentation in broth culture, but are not crucial for viability. Moreover, Chs1p seems to contribute to growth of *phr1*Δ, although this aspect was not further explored in this study. Our results indicate that a complex interplay among the different isoforms buffers the damage to the hyphal cell wall. These results can be reconciled by taking into account that different isoforms produce different types of chitin fibrils not all of which may be equally effective in reinforcing the lateral wall. The recruitment of other *CHS* isoforms during HWS reveals a difference between *C. albicans* and *S. cerevisiae.* In *S. cerevisiae* the absence of Chs3p does not lead to chitin deposition by other isoforms, namely the Class I chitin synthase Chs1p (equivalent to Chs2p and Chs8p) despite the fact that *ScCHS1* is always up-regulated by cell wall stress [57, 64].

The *phr1*Δ mutation combined with deletion of *CHSx* genes produced a more severe phenotype during hyphal growth on agar-solidified media. *PHR1* was synthetic lethal with *CHS3* at the onset of germination on M199-pH 7.5. At pH 8, loss of *CHS8* alone, or in combination with *CHS2* inactivation, produced cell death or a very strong lysis phenotype of germinated cells, respectively. Thus, the requirement for Chs3p and Chs8p functions occurs at different stages in the transition of single cells to filamentous growth. On Spider medium, *PHR1* was synthetic lethal with *CHS3* or *CHS8* only at pH 8. Therefore, nutrient-rich growth media, such as M199, represent a more restrictive condition for *phr1*Δ cells whereas a slow-filamentation medium, such as Spider, allows adaptation, provided HWS is not too severe. These results clearly demonstrate that *CH*S3 and *CHS8* have unique roles in cell wall chitin synthesis, and one cannot compensate for the other(s). Moreover, their roles are not evident by simply assessing total chitin content.

A recent report demonstrated for the first time that class I chitin synthases (Chs2p and Chs8p) in *C. albicans* play a role in maintaining cell integrity during early polarized bud and hyphal growth and immediately following septation events in hyphae and in the presence of stress [65]. This role is supported by the dynamic localization of these enzymes [65]. This may explain why in a condition of intense polarization such as the Y-H transition the lack of glucan remodeling makes the activity of the otherwise dispensable *CHS8* physiologically crucial.

Since Chs8p and Phr1p localize to the hyphal septum, we also postulate that loss of Phr1p function at this site is highly harmful for *C. albicans*. Our model predicts that in the absence of Chs8p and Phr1p, more Chs3p is recruited to the septum to reinforce the cell wall during filamentation. This may explain the decrease in CW-staining of lateral walls and the maintenance of an intense signal at the septum in the *chs8*Δ *phr1*Δ mutant (Fig. 3a). Experiments using fluorescent-tagged versions of Chs proteins will be necessary to test this model.

Finally, it is well-known that Chs3p is regulated primarily at a post-transcriptional level such as recruitment to the plasma membrane from intracellular compartments and recycling (ER, *Trans*-Golgi network, endosomal system) and regulation of its location by phosphorylation [49]. At 5 h, the level of the *CHS7* transcript in *phr1*Δ cells is higher than in the wild type suggesting that Chs3p folding and export from ER could be crucial in the response to HWS.

### Other functions affected by HWS

Finally, we demonstrated that the block in morphogenesis brings about an accelerated entry into S-phase and G_2_. In *C. albicans,* cells enter S-phase when germ tubes reach a critical length or size. Stabilization of the G_1_ cyclin and delayed accumulation of the mitotic cyclins Clb2 and Clb4 occurring during germ tube formation suggests an extended G_1_ phase with respect to yeast cells [66–68]. The mechanism regulating this seems to be altered or negated in the presence of *phr1*Δ defects which block the hyphal morphogenic program. Moreover, in the mutant, genes involved in rRNA processing, maturation and transport are repressed at 1 h. This response could be a consequence of the morphogenetic defect as an early repression of ribosome biogenesis genes was also detected in the block of hyphae formation by farnesol [39].

Interestingly, the cyclin genes *PCL1*, *PCL2* and *CCN1*, a homolog of *CLB3* in *S. cerevisiae*, normally repressed in cells induced to make hyphae, are up-regulated both in *phr1*Δ and in farnesol-arrested hyphae (Table 2 and [39]). Also, the cyclin-dependent kinase *CDC28* is up-regulated at all three time points in the *phr1*Δ mutant. The high RF at 5 h for *CCN1, CDC28, GIN4* suggests that the complex of Ccdc28p-Ccn1p, that associates with the septins and phosphorylates Cdc11p after a previous phosphorylation requiring the septin-associated Gin4p [69], may persist in the mutant and possibly involved in preventing catastrophic effects on the nuclear division cycle. Future studies will address the relations between the block of morphogenesis and cell cycle-regulated events under hyphal growth conditions.

## Conclusions

The glucan remodeling activity catalyzed by Phr1p is fundamental for the progression of hyphal growth and its absence modifies genome-wide expression profiles to trigger a reinforcement of the cell surface and changes in several functions affecting metabolism and transport, cell cycle and DNA replication, transcription and stress response in order to restore integrity and homeostasis. Genes that are differentially modulated compared to the wild type help explain some phenotypic traits typical of the mutant such as the defect of adhesion, besides providing evidence of new functions with potentially important roles in the adaptive response to HWS. One of these genes is *FLC2* encoding a putative calcium transporter that in *S. cerevisiae* is involved in the release of calcium from intracellular stores in response to hypotonic shock [37]. Our findings also revealed a novel essential role of *CHS8* in protecting cell integrity during growth on agar plates in condition of very severe HSW.

## Methods

### Strains and growth conditions

The strains used in this work are listed in Table 4. To induce hyphal development, stationary phase cells from an overnight culture in YPD (1 % yeast extract, 2 % peptone, 2 % glucose)-150 mM HEPES pH 6.0 incubated at 30 °C were collected by centrifugation. The pre-culture medium was buffered at pH 6 to limit the cellular content of Phr2p, as at this pH the expression of *PHR2* is extremely low but sufficient to prevent manifestation of the phenotype of *phr1*Δ cells [19]. Cells were suspended at an initial OD_600_ of about 0.25 in pre-warmed M199-150 mM HEPES buffered at pH 7.5 and supplemented with 1.9 % glucose, and incubated under agitation at 37 °C. For Ura^−^ strains, 25 μg/ml uridine was added to YPD. The formation of hyphae was monitored every 30 min.

**Table 4:** *Candida albicans* strains used in this work

Strain	Parental Strain	Genotype^a^	Source or reference
CAF2-1	SC5314	*URA3/ura3*∆::*λimm434*	[71]
CAI4	CAF2-1	*ura3* ∆::*λimm434*/*ura3*∆:: *λimm434*	[71]
CAF3-1	CAF2-1	*ura3* ∆::*λimm434*/*ura3*∆:: *λimm434*	[71]
CAS8	CAF3-1	*phr1*∆*::hisG/phr1*∆	[20]
CAI10	CAF3-1	*URA3/ura3* ∆::*λimm434*	[71]
CAS10	CAS8	*phr1*∆*::hisG/phr1*∆ *URA3/ura3* ∆*::λimm434*	[20]
C155	CAI4	*chs2*∆::*hisG/chs2*∆::*hisG*	[45]
FP155-14-21	C155	*phr1*∆*::hisG-URA3-hisG/phr1*∆*::NAT1 chs2∆::hisG/chs2∆::hisG*	This work
FP155-19-21	C155	*phr1*∆*::hisG-URA3-hisG/phr1*∆*::NAT1 chs2∆::hisG/chs2∆::hisG*	This work
Myco3	Myco4	*chs3*∆::*hisG/chs3*∆::*hisG*	[46]
FP3-19-211	Myco3	*phr1*∆*::hisG-URA3-hisG/phr1*∆*::NAT1 chs3∆:: hisG/chs3∆::hisG*	This work
FP3-118-25	Myco3	*phr1∆::hisG-URA3-hisG/phr1∆::NAT1 chs3∆:: hisG/chs3∆::hisG*	This work
NGY128	CAI4	*chs8*∆::*hisG/chs8*∆::*hisG*	[44]
FP128-19-21	NGY128	*phr1*∆*::hisG-URA3-hisG/phr1*∆*::NAT1 chs8∆::hisG/chs8∆::hisG*	This work
FP128-111-21	NGY128	*phr1*∆*::hisG-URA3-hisG/phr1*∆*::NAT1 chs8∆:: hisG/chs8∆::hisG*	This work
NGY138	CAI4	*chs2∆::hisG/chs2∆::hisG chs8∆:: hisG/chs8∆::hisG*	[44]
FP138-16-22	NGY138	*phr1∆::hisG-URA3-hisG/phr1∆::NAT1 chs2∆::hisG/chs2∆::hisGchs8∆::hisG/chs8∆::hisG*	This work
FP138-114-21	NGY138	*phr1∆::hisG-URA3-hisG/phr1∆::NAT1 chs2∆::hisG/chs2∆::hisGchs8∆::hisG/chs8∆::hisG*	This work

^a^all strains apart from CAF2-1, CAI10 and CAS10 are homozygous for the *ura3* Δ::*λimm434* mutation [71]

For the filamentation plate assay, M199-150 mM HEPES, supplemented with 2 % agar, was adjusted to the desired pH and supplemented with 100 μg/ml^−1^ uridine while Spider was prepared as described [70] and 0.8 M sorbitol was included if required. Stationary phase cells from an overnight culture in YPD+ Uri -pH 6 at 30 ° C were diluted to 10^3^/cells ml in buffered liquid medium. 100 or 150 μl of the cell suspension was spread and plates were incubated at 37 °C for 24 to 72 hours. Plates were observed using a Leica MZ6 stereomicroscope.

### Mutant strain construction

Strains C155 (*chs2*Δ/*chs2*Δ), NGY128 (*chs8*Δ/*chs8*Δ), Myco3 (*chs3*Δ/*chs3*Δ), and NGY138 (*chs2*Δ/*chs2*Δ *chs8*Δ/*chs8*Δ) were kindly provided by Prof. Neil Gow (Aberdeen Fungal Group, University of Aberdeen, UK) and transformed with *Hin*dIII and *Pvu*II digested DNA of plasmid pSMS23 to delete one allele of *PHR1* [20, 71]. Cell transformation was performed as described [72]. Ura^+^ transformants were selected on SD agar. Linkage of the transforming DNA with the *PHR1* locus was verified by PCR amplification using primers PHR1-N7 and hisG3'P for the 5’-end and primers hisG-forward and PHR1rev(+1840) for the 3’-end. The primers used in this work are listed in Additional file 8. Deletion of the second allele was achieved by transformation with *Btg*ZI and *Pvu*II digested DNA of plasmid pLit-PHR1-1NAT DNA. Plasmid pLit-PHR1-1NAT was constructed by cloning a 2.5 kb *Eco*RI-*Hin*dIII fragment from a genomic clone of *PHR1* [73] into the same sites of pLITMUS38 (New England Biolabs). The resulting plasmid pLit38PHR1-1 was digested with *Sac*II and *Kpn*I, which removed nucleotides +167 to +853 of the *PHR1* coding region, and ligated with a 1265 bp *Sac*II-*Kpn*I fragment containing the nourseothricin resistance gene, *NAT1*, isolated from plasmid pJK795 [74]. Transformed cells were selected on SD medium containing 450 ug/ml nourseothricin. Linkage to the *PHR1* locus was tested with PCR primers PHR1-N7 and NAT1_5’link_rev for the 5’ end and NAT1-3'link_fwd and PHR1rev(+1840) for 3’-linkage. Absence of a wild type allele was verified with primers PHR1-N7 and PHR1-rev(+1840). Two independent *phr1*Δ null mutants were analyzed in each *chsx*Δ mutant background.

### DNA microarray analysis

At 0, 1, 3 and 5 hours after the shift of stationary phase cells to the hyphal growth inducing medium, cells (equivalent to 200 units of OD_600_) were collected by filtration on a Millipore 0.45 μm filter disk. The filter was removed, transferred to a tube and the cells suspended in 1 ml of ice-cold H_2_O. The washed cells were quickly frozen in liquid nitrogen and stored at −80 °C until use. Cell samples were collected from two independent cultures.

Total RNA was extracted using the RNeasy Midi Kit (QIAGEN). Cell pellets were thawed and suspended in 600 μl of RTL buffer. After addition of an equal volume of cold, sterile glass beads, cells were broken by 5 cycles of 1 min agitation in a BeadBeater (Biospec) at 4 °C and 1 min on ice. Total RNA was isolated from two independent experiments. RNA concentration and quality were assessed by spectrophotometric measurements and denaturing agarose gel electrophoresis. RNA samples were processed for further analysis at the Microarray Facility, Washington University at St. Louis, Mo U.S.A. (currently GTAC, Genome Technology Access Center).

### DNA microarray hybridization

RNA integrity and quality were validated on an Agilent 2100 Bioanalyzer and reverse transcribed into cDNA in the presence of Cy3/Cy5 labeled dNTPs using the 3DNA array 350 detection system (Genisphere, Hatfield, PA). For each strain, data from 1, 3 and 5 h were compared to time zero. To exclude any influence due to the type of fluorophore used, the labeling of the samples was also inverted (dye-swap). For two biological replicas, a total of 24 competitive hybridizations (1 h, 3 h or 5 h versus time zero) were performed (12 for each strain). DNA oligonucleotide microarrays represented 6,346 of the 6,354 predicted ORFs in the annotated *C. albicans* genome assembly 19. Probes consisted of unique ORF-specific 70-mer oligonucleotides, oligonucleotide sequences were selected using an ArrayOligoSelector software and were obtained from Illumina (San Diego, CA). Each probe was spotted three times per slide, thus 19,308 spots were present on each array plus 10 genes from *Arabidopsis thaliana* as specificity controls. A two-step protocol was used for hybridization (3DNA array 350 detection system). First, oligonucleotide arrays were hybridized to the cDNA probes in 2× formamide-based hybridization buffer overnight at 43 °C and washed in 2× SSC (1× SSC is 0.15 M NaCl plus 0.015 M sodium citrate)-0.2 % sodium dodecyl sulphate (SDS) according to the manufacturer's protocol. Fluorescent Cy3- and Cy5-capture reagents were combined in hybridization buffer and added to each array, which were incubated and washed again as described above. Slides were scanned immediately after hybridization on a ScanArray express HT scanner (Perkin-Elmer) to detect Cy3 and Cy5 fluorescence. The laser power was kept constant, and photomultiplier tube (PMT) values were set for optimal intensity with minimal background. An additional scan was done for each slide with the PMT such that <1 % of the elements are saturated in order to characterize spots which were saturated at the higher PMT setting. Gridding and analysis of images was performed with ScanArray software express V2.0 (Perkin-Elmer).

### Data processing and statistical analysis

Microarray data were deposited at GEO database (GSE51064). Microarray image analysis was done using the analysis software from Partek Incorporated. Intra-array data processing was performed basically as previously described [58], adapting the protocol to the microarray design used in our work. Flagged spots and spots with an average intensity minus background below the mean of the background for all the non-flagged spots in any of the channels (Cy3 or Cy5) were not retained for further analysis. Within this group the spots, showing in one channel a value of intensity minus background higher than 5 times the mean of the background for all spots in that channel, were recovered. This group could correspond to potential on/off genes. Genes that did not have at least 2 valid replicates (the microarrays include 3 spots per ORF) or exceeded the intensity average by more than 1.5 S.D. were also discarded. Next, filtered data for each microarray were normalized using a Lowess normalization method performed with the web-software Babelomics [75]. Finally, taking into account that for each experimental condition we performed four competitive hybridizations (2 biological replicates and 2 dye-swap), the average (arithmetic mean) of expression ratios was calculated accepting only those genes with at least three data available, and one-sample Limma analysis and False Discovery Rate (FDR, using a *p*-value ≤ 0.05) for multiple testing correction were applied [76, 77] using the software Babelomics. The cutoff to consider transcriptional induction or repression with respect to time zero was a ratio ≥ 2 and ≤0.5, respectively.

For the comparison of the transcriptional profile of the *phr1*Δ mutant versus the wild type strain, we calculated a Relation Factor (RF, ratio of the expression ratios) that measures the effect of the mutation on the transcript induction or repression observed in the wild-type strain. We performed an additional statistical test using Limma analysis and False Discovery Rate (FDR, using a *p*-value ≤ 0.05) comparing the two groups of expression ratios (mutant vs wild type) for each time point analyzed. After this analysis, we selected only those genes showing statistically significant RFs ≤ 0.5 or ≥ 2 in order to identify genes displaying relevant altered expression profiles for both strains. Genes were grouped in two classes depending on their RF values and expression ratios.

Principal Component analysis was performed using the software Partek. Hierarchical cluster analysis was performed using Pearson correlation (average linkage) and the Multiple Viewer Experiment (MeV) software package developed by TIGR [78].

### Verification of differential gene expression using qRT-PCR

Primer sequences are shown in Additional file 8. Total RNA was extracted at time zero, 3 and 5 hours after induction of hyphal growth. On-column DNase digestion was performed according to the manufacturer’s instructions (QIAGEN). First strand cDNAs were synthesized from 1.6 μg of total RNA in 20 μl final volume, using the Reverse Transcription System A3500 (Promega). As a test for residual genomic DNA contamination, reactions were performed in the absence of reverse transcriptase. Real time quantitative PCR reactions were carried out in a Bio-Rad IQ5 instrument. Each PCR reaction contained 5 μl of diluted cDNA, 7.5 μl of SsoFast™EvaGreen^®^Supermix with low ROX (Bio-Rad), 1.5 μl of oligos (final concentration each 0.5 μM) and 1 μl nuclease-free H_2_O. Triplicates of all reactions were analysed. For quantification, the abundance of each transcript during induction conditions was determined relative to the standard *TDH3* transcript, as indicated in other reports [79, 80]. Final data on relative gene expression between the two conditions (sample from time 3 or 5 h with respect to time zero of each strain) and from two independent biological replicates were calculated according to the 2^-ΔΔ*C*T^ method [81].

### Microscopy

For CW staining, cells (about 1–2 OD_600_) were washed with 0.5 ml of dH_2_O and suspended in 0.5 ml of CW (0.1 mg/ml dH_2_O). After 5 min, the sample was washed twice with 1 ml of dH_2_O and examined by fluorescence microscopy. DAPI staining was performed as previously described [25]. Fungal viability was assessed by methylene blue (MB) staining, which identifies metabolically inactive or membrane compromised cells. Viable cells appear colorless while dead cells are blue. Cells were collected by centrifugation and suspended in the same volume of MB solution (MB 0.2 g/l, KH_2_PO_4_ 27.2 g/l, K_2_HPO_4_ 0.071 g/l, pH 4.6 stored at 4 °C) and incubated for 20 min at RT prior to examination by bright-field microscopy.

### Broth microdilution assay

Sensitivity to the Chs1p inhibitor RO-09-3143 was tested in a microdilution assay performed according to the NCCLC guidelines M27-A2 using an inoculum size of 5 x 10^5^/ml as previously described [82]. Briefly, cells were grown to stationary phase in YPD-HEPES buffered at pH 6 at 25 °C. In a well of a 96-well microtiter plate, 100 μl of a suspension containing 10^6^ cells/ml in M199-HEPES, pH 7.5 supplemented with 100 μg/ml of uridine, was added to an equal volume of medium containing RO-09-3143 (kindly donated by Roche) dissolved in DMSO. Two-fold serial dilutions of the inhibitor were made to achieve a range of concentrations from 0.012 μM to 25 μM. All determinations were made in duplicate. Control wells contained DMSO with no drug. The plates were incubated at 37 °C and inspected at 24 and 48 h. The effect of the treatment was evaluated both visually and also by reading the turbidity with a Tecan Infinite F200 PRO microtiter plate reader.

### Enzymatic assay of the chitin content

Cells (about 150–200 of total OD_600_) were collected by centrifugation, suspended in 6 ml of H_2_O, and divided into four equal aliquots, two of which were used to determine the cell dry weight. The other two were centrifuged and the pellets stored at −20 °C for later chitin determinations. Total cellular chitin was measured by an enzymatic method, as described previously [83]. Washed cells (about 100 mg wet weight) were suspended in 1 ml 6 % KOH and incubated at 80 °C for 90 min. After cooling, 100 μl of glacial acetic acid were added and samples were centrifuged for 15 min at 13,000 x g. The pellet, containing the alkali-insoluble material, was washed twice with 50 mM phosphate buffer, pH 6.3 and suspended in 1 ml of the same buffer containing 1.7 mg of *Serratia marcescens* chitinase (Sigma). After 2 h at 37 °C, 400 μl were transferred to a new tube and 25 μl of *Helix pomatia* β-glucuronidase (Roche) were added. Samples were incubated for 1 h at 37 °C. After 1 min-incubation at 100 °C, samples were centrifuged and the supernatants were saved. The amount of GlcNAc was determined with the Morgan-Elson reaction. To 100 μl of sample, 150 μl of H_2_O were added. After 1 min-incubation at 100 °C and addition of 250 μl of 0.27 M potassium tetraborate, pH 9, samples were boiled again for 8 min. After cooling, 3 ml of Ehrlich-solution (stock solution 10 times concentrated was prepared by mixing 10 g of p-dimethylaminobenzaldehyde (Sigma) in 12.5 ml of 10 N HCl and 87.5 ml of glacial acetic acid) was added and the samples were incubated for 40 min at 37 °C. The absorbance at 585 nm was measured and compared with a standard curve ranging from 0 to 1 mg of GlcNac. The micrograms of GlcNac were normalized to the milligrams of dry weight of cells.

### Protein extracts and Western analysis

For the analysis of Phr1p levels, total extracts were prepared as previously described [16]. For the analysis of the activation of MAP kinases, a quick TCA precipitation method was used. Briefly, cells (10^8^) were collected and suspended in 2 ml of 20 % TCA and either processed or stored at −20 °C. After centrifugation at 20,000 RCF for 3 min, the pellet was suspended in 100 μl of 20 % TCA. Cells were broken mechanically by vortexing for 4 min after addition of glass beads. Then, 200 μl of 5 % TCA were added and vortexing was prosecuted for another 3 min. The liquid phase was aspirated and transferred to a new tube. After centrifugation at 960 RCF for 10 min, the supernatant was discarded while the pellet was let to dry and then suspended in SDS-sample buffer [0.0625 M Tris–HCl, pH 6.8, 2.3 % (w/v) SDS, 5 % (v/v) β-mercaptoethanol, 10 % (w/v) glycerol and 0.01 % bromophenol blue (BBF)] and 50 μl of Tris base 1 M were added to each sample. Samples were incubated at 37 °C for 20 min. Extracts were adjusted to have equivalent amount of proteins on each lane. Alternatively, SB-minus buffer [0.0625 M Tris–HCl, pH 6.8 and 2.3 % SDS] was used and protein concentration was determined by BCA protein assay (Pierce). Samples containing equivalent amount of proteins were adjusted by addition of β-mercaptoethanol, glycerol and BBF prior to loading the gels. Proteins were separated on 10 % SDS-PAGE gels and then blotted to nitrocellulose. The anti-phospho-p44/p42 MAPK (Thr^202^ /Tyr^204^) antibody and the Phospho-p38 MAP kinase (Thr^180^/Tyr^182^) antibody (Cell Signaling technology, U.S.) were used to detect the dual phosphorylated forms of Mkc1p, Cek1p MAP kinases and Hog1 MAP kinase, respectively. The anti-*C. albicans* Mkc1p and anti-Cek1p antibodies were kindly provided by J. Plà and R. Alonso-Monge (Universidad Complutense de Madrid). Hog1p was detected using y-215 antibody (Cell Signaling technology, U.S.) at a dilution of 1:500. Anti-actin mouse mAb was from Millipore. Peroxidase-conjugated anti rabbit or anti-mouse antibodies were from Sigma.

### Flow cytometric analysis of DNA

Stationary phase cells were shifted to M199-pH 7.5 at 37 °C at an initial density of 0.25 OD_600_. At different time intervals after induction of hyphal growth, 1 OD_600_ of cells were mildly sonicated, collected by centrifugation, fixed in ice-cold 70 % ethanol and stored at 4 °C. The samples were suspended in 0.5 ml of RNase A (1 mg/ml in 50 mM Tris–HCl pH 7.5) and incubated at 37 °C overnight. The samples were centrifuged and suspended in 0.5 ml of Proteinase K (2 mg/ml in 50 mM Tris–HCl pH 7.5). After 2 h at 42 °C, the samples were centrifuged, suspended in FACS buffer (200 mM Tris–HCl pH 7.5, 200 mM NaCl, 78 mM MgCl_2_) and stored at 4 °C. At the time of the analysis, 100 μl of sample was added to 1 ml of Sytox Green 1x, a nucleic acid stain (diluted 1:5000 in 50 mM Tris–HCl pH 7.5; Invitrogen), subjected to a brief sonication and analyzed with a FACScan flow cytofluorimeter (BD Biosciences, San Jose, Ca). We analyzed 10^4^ cells for each sample.

## Abbreviations

CW, calcofluor white; CWI, cell wall integrity; HOG, high-osmolarity glycerol; HWS, hyphal wall stress; MB, methylene blue.

## Additional files

Additional file 1: Transcriptional profile of wild type and *phr1*Δ cells during induction of hyphal growth. (XLSX 735 kb) Additional file 2: Class 1: Transcripts that are more abundant in the *phr1*Δ mutant than in the wild type. (XLSX 45 kb) Additional file 3: Class 2: Transcripts that are less abundant in the *phr1*Δ mutant than in the wild type. (XLSX 39 kb) Additional file 4 Comparison of data from microarray and qRT-PCR analyses. (DOC 41 kb) Additional file 5: Effects of Chs1p inhibition on mutant viability. Samples from a representative microdilution assay as described in Fig 4. Cells were aspirated from wells of a microtitre plate containing the indicated concentrations (μM) of RO-09-3143, stained with MB and examined microscopically. Scale bar, 10 μm. (TIF 8871 kb) Additional file 6: Comparison of colonies on solid M199-pH 8. Colonies of the indicated strains were photographed after 3 days on M199-pH 8 at 37 °C. The figure shows representative images from different independent experiments (*n* = 4). The bar is equal to 1 mm. (TIF 2893 kb) Additional file 7: Comparison of colonies on Spider medium pH 7.5 or pH 8. Spider-150 mM HEPES supplemented with uridine were buffered at pH 7.5 or pH 8 and plates were incubated for 7 days at 37 °C. The figure shows representative images from different independent experiments (*n* = 3). (TIF 4371 kb) Additional file 8: Oligonucleotides used in this work. (DOC 33 kb)
