# Supplementary material for: Genomic and functional analyses unveil the response to hyphal wall stress in Candida albicans cells lacking β(1,3)-glucan remodeling
Source: BMC Genomics. 2016 Jul 2;17:482. doi: 10.1186/s12864-016-2853-5 (PMC4942948; doi:10.1186/s12864-016-2853-5)
Supplement: Additional file 4 — Comparison of data from microarray and qRT-PCR analyses. (DOC 41 kb) [file 12864_2016_2853_MOESM4_ESM.doc]

**Additional file 4.** Comparison of data from microarray and qRT-PCR analyses.

| **Gene** | **Time** | **DNA Microarray** | | | **qRT-PCR** | | |
| --- | --- | --- | --- | --- | --- | --- | --- |
|  | **3 hr** | **WT^a^** | ***phr1^b^*** | **RF^c^** | **WT fold-change^d^** | ***phr1* fold-change^e^** | **RF** |
| *CRH11* |  | 1.2 | 5.1 | 4.2 | 2.5 ± 0.3 | 7.2 ± 0.2 | 2.8 ± 0.1 |
| *GFA1* |  | 2.3 | 4.8 | 2.1 | 1.4 ± 0.5 | 3.7 ± 0.8 | 2.8 ± 0.4 |
| *CHS8* |  | 3.0 | 5.9 | 2.0 | 2.1 ± 0.3 | 7.2 ± 0.1 | 3.5 ± 0.2 |
| *PGA23* |  | 0.2 | 4.2 | 22.2 | 0.2 ± 0.01 | 5.6 ± 0.5 | 29.2 ± 0.1 |
|  | **5 hr** |  |  |  |  |  |  |
| *CRH11* |  | 2.3 | 7.6 | 3.3 | 2.1 ± 0.2 | 6.6 ± 2.7 | 3.1 ± 0.4 |
| *GFA1* |  | 3.3 | 7.6 | 2.3 | 0.6 ± 0.4 | 2.1 ± 0.5 | 3.3 ± 0.6 |
| *CHS8* |  | 2.6 | 11.1 | 4.3 | 1.6 ± 0.2 | 4.5 ± 0.9 | 2.9 ± 0.2 |
| *PGA23* |  | 0.2 | 10.2 | 65.6 | 0.1 ± 0.01 | 7.3 ± 0.4 | 93.1 ± 0.1 |

^a^ and ^b^ Expression ratio of each gene at the indicated time compared to time zero in the same strain.

^c^ RF, Relation Factor, ratio of the expression ratios or fold-change MUT/WT

^d^ and ^e^ Fold-change of the transcript level of each gene at the indicated time with respect to time zero.
