# Supplementary figures and images for: Genomic and functional analyses unveil the response to hyphal wall stress in Candida albicans cells lacking β(1,3)-glucan remodeling
Source: BMC Genomics. 2016 Jul 2;17:482. doi: 10.1186/s12864-016-2853-5 (PMC4942948; doi:10.1186/s12864-016-2853-5)

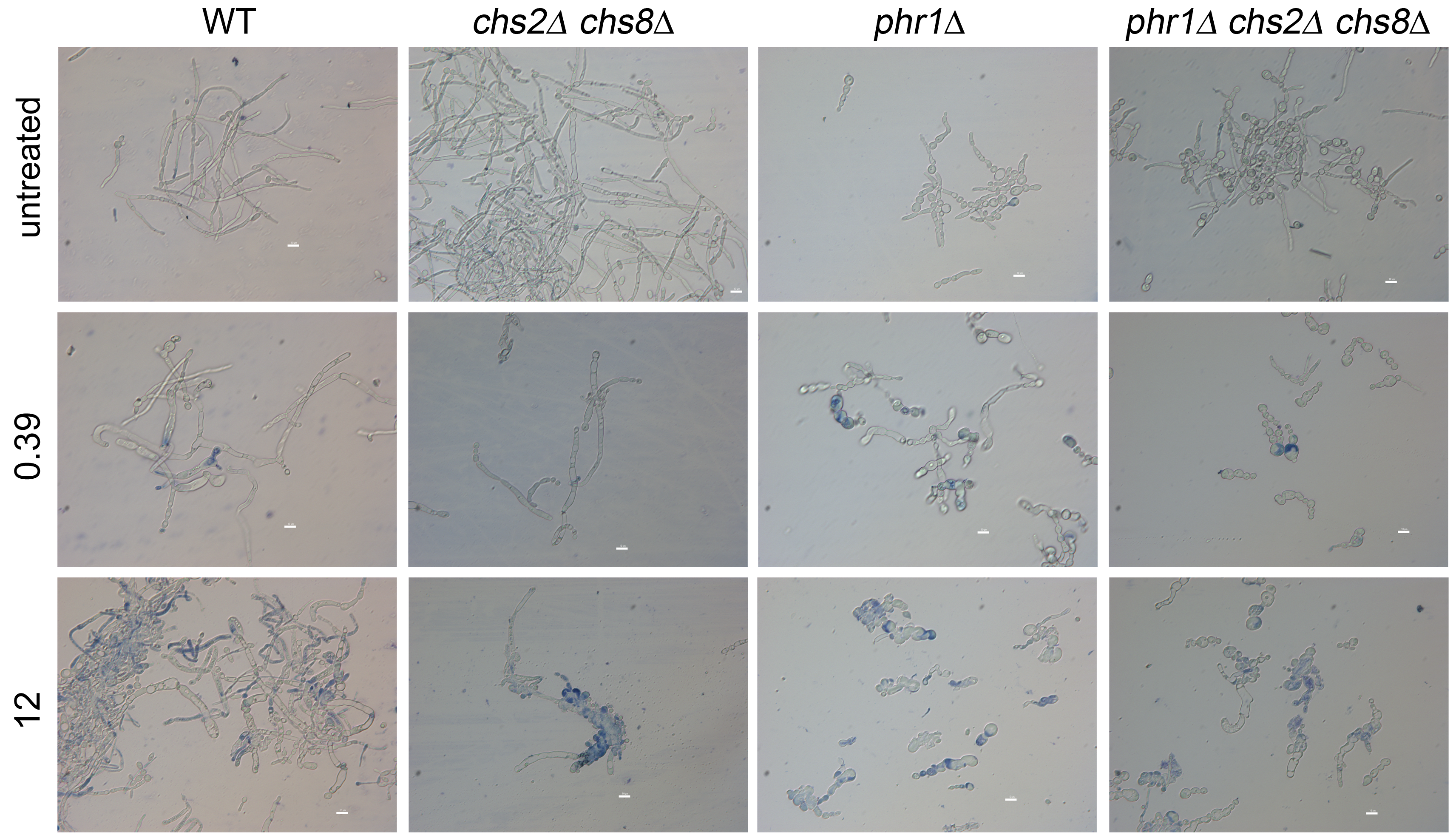

Supplement: Additional file 5: — Effects of Chs1p inhibition on mutant viability. Samples from a representative microdilution assay as described in Fig 4. Cells were aspirated from wells of a microtitre plate containing the indicated concentrations (μM) of RO-09-3143, stained with MB and examined microscopically. Scale bar, 10 μm. (TIF 8871 kb) [file 12864_2016_2853_MOESM5_ESM.tif]

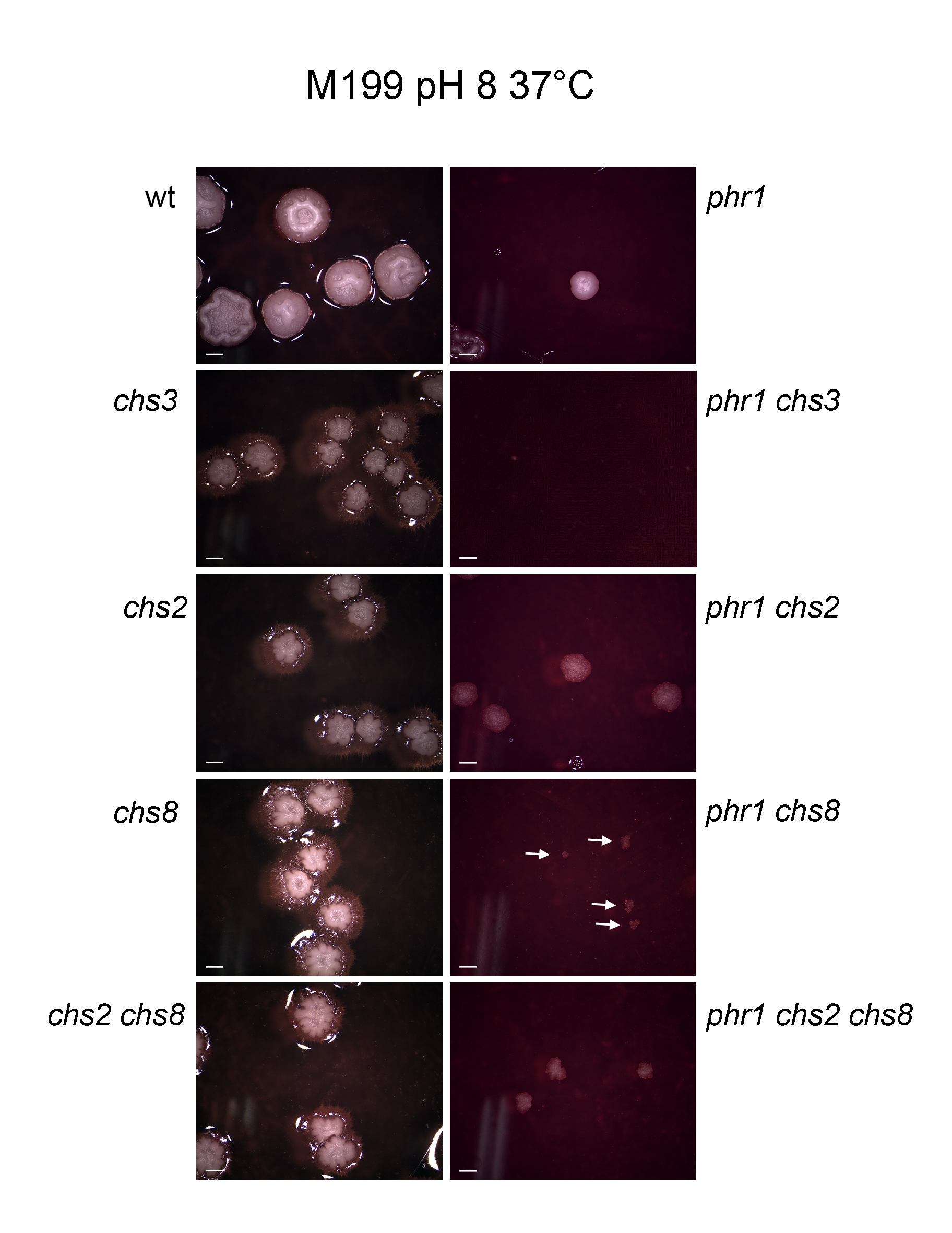

Supplement: Additional file 6: — Comparison of colonies on solid M199-pH 8. Colonies of the indicated strains were photographed after 3 days on M199-pH 8 at 37 °C. The figure shows representative images from different independent experiments (n = 4). The bar is equal to 1 mm. (TIF 2893 kb) [file 12864_2016_2853_MOESM6_ESM.tif]

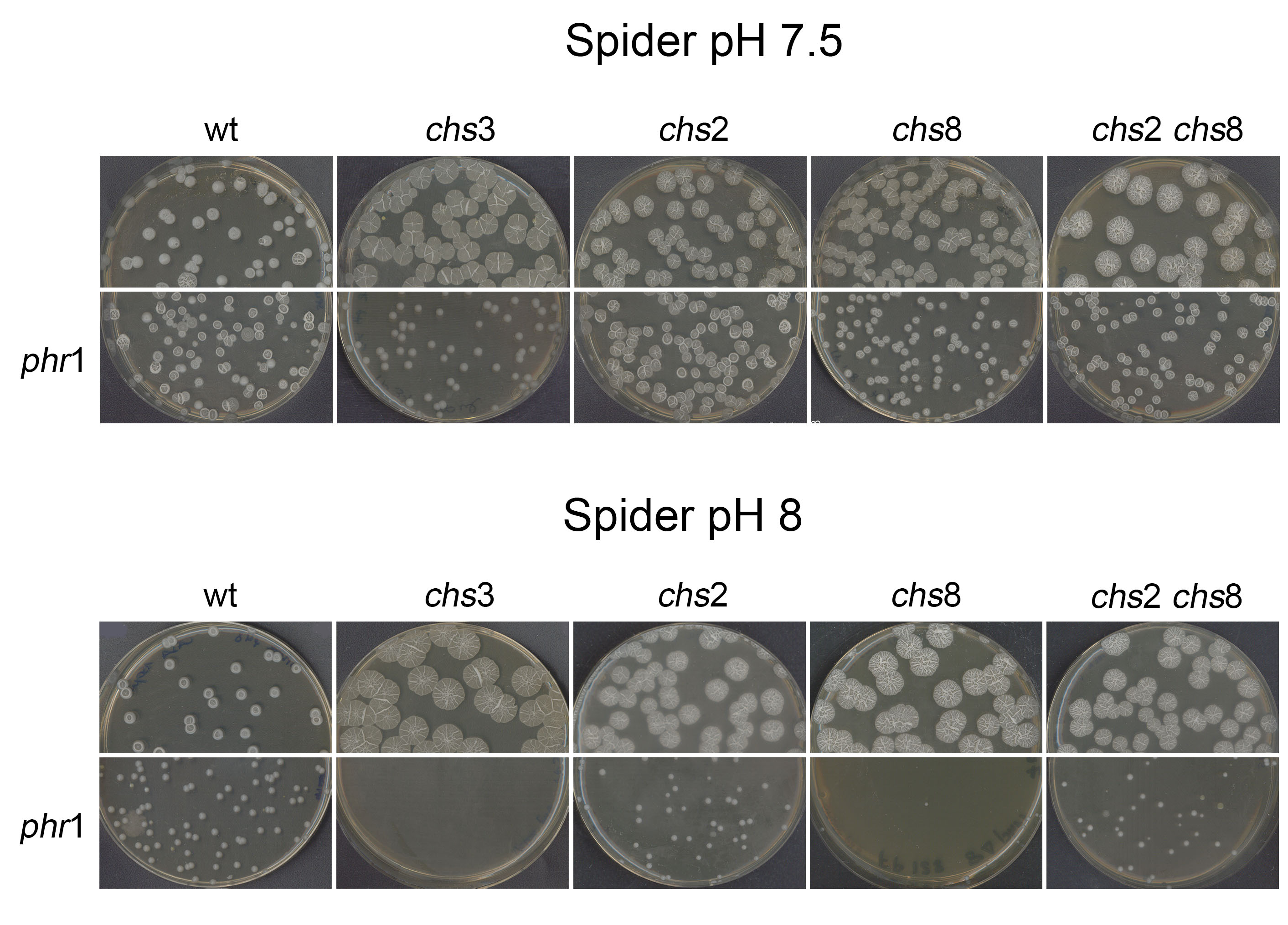

Supplement: Additional file 7: — Comparison of colonies on Spider medium pH 7.5 or pH 8. Spider-150 mM HEPES supplemented with uridine were buffered at pH 7.5 or pH 8 and plates were incubated for 7 days at 37 °C. The figure shows representative images from different independent experiments (n = 3). (TIF 4371 kb) [file 12864_2016_2853_MOESM7_ESM.tif]
