# Supplementary material for: Genomic and functional analyses unveil the response to hyphal wall stress in Candida albicans cells lacking β(1,3)-glucan remodeling
Source: BMC Genomics. 2016 Jul 2;17:482. doi: 10.1186/s12864-016-2853-5 (PMC4942948; doi:10.1186/s12864-016-2853-5)
Supplement: Additional file 8: — Oligonucleotides used in this work. (DOC 33 kb) [file 12864_2016_2853_MOESM8_ESM.doc]

| **Primer** | **Sequence** |
| --- | --- |
| **Primers for strain construction** |  |
| PHR1-N7 | 5’-CCATGGCCTCCTTAGATCAAATCATTCC-3’ |
| hisG3'P | 5’-GCCAAGCGCTTTCAGTTTCTC-3’ |
| hisG-forward | 5’-CCTCATATCCGCACCTCCTC-3’ |
| PHR1rev(+1840) | 5’-CACCAATTGAGAAACTTAGGGAT-3’ |
| NAT1_5’link_rev | 5-GAGCCGTAATTTTTGCTTCG-3’ |
| NAT1-3'link_fwd | 5’-TGGTCGCTATACTGCTGTCG-3’ |
| **Primers for Q-RT-PCR** |  |
| CRH11-F | 5'-CTACCGTTACCAGTTCTTCG-3' |
| CRH11-R | 5'-CAGTGGAGGCAGTACTTGAA-3' |
| GFA1-F | 5'-ATCTGCTGGGGAATCTACTG-3' |
| GFA1-R | 5'-GCAGAATCTGGTTGTTCG-3' |
| CHS8-F | 5'-GGTAGTGGTGGAATACATGC-3' |
| CHS8-R | 5'-ATCTCTTCACCAGTGTCCAG-3' |
| TDH3-F | 5'-GGTTTCGGTAGAATCGGTAG-3' |
| TDH3-R | 5'-CACCCTTGTATCTACCGTGA-3' |
| PGA23-F | 5'-GGAAGCTTAGTTGGTGGTCT-3' |
| PGA23-R | 5'-TCTAGATCCTGAACCACTGC-3' |

**Additional file 8. Oligonucleotides used in this work.**
